# HER2 is not a cancer subtype but rather a pan-cancer event and is highly enriched in AR-driven breast tumors

**DOI:** 10.1186/s13058-018-0933-y

**Published:** 2018-01-30

**Authors:** Anneleen Daemen, Gerard Manning

**Affiliations:** Bioinformatics & Computational Biology, Genentech, Inc, 1 DNA Way, MS444a, South San Francisco, CA 94080 USA

**Keywords:** Breast cancer, Cancer, Amplification, ERBB2, Genomic characterization, PAM50, Molecular apocrine, HER2-targeted treatment

## Abstract

**Background:**

Approximately one in five breast cancers are driven by amplification and overexpression of the human epidermal growth factor receptor 2 (HER2) receptor kinase, and HER2-enriched (HER2E) is one of four major transcriptional subtypes of breast cancer. We set out to understand the genomics of HER2 amplification independent of subtype, and the underlying drivers and biology of HER2E tumors.

**Methods:**

We investigated published genomic data from 3155 breast tumors and 5391 non-breast tumors.

**Results:**

HER2 amplification is a distinct driver event seen in all breast cancer subtypes, rather than a subtype marker, with major characteristics restricted to amplification and overexpression of HER2 and neighboring genes. The HER2E subtype has a distinctive transcriptional landscape independent of HER2A that reflects androgen receptor signaling as replacement for estrogen receptor (ER)-driven tumorigenesis. HER2 amplification is also an event in 1.8% of non-breast tumors.

**Conclusions:**

These discoveries reveal therapeutic opportunities for combining anti-HER2 therapy with anti-androgen agents in breast cancer, and highlight the potential for broader therapeutic use of HER2 inhibitors.

**Electronic supplementary material:**

The online version of this article (10.1186/s13058-018-0933-y) contains supplementary material, which is available to authorized users.

## Background

Transcriptional profiling has enabled the classification of many cancers into distinct gene expression subtypes, allowing improved diagnosis and treatment selection. Breast cancer has four well-established, transcriptional subtypes in the prediction analysis of microarray 50 (PAM50) scheme: luminal A, luminal B, basal-like, and human epidermal growth factor receptor 2 (HER2)-enriched (HER2E) [1]. These subtypes overlap with immunohistochemical (IHC) staining of three protein markers, estrogen receptor (ER), progesterone receptor (PR) and HER2, supplemented with in situ hybridization (ISH) of HER2. The luminal A and B subtypes are enriched for ER+ tumors and the basal-like subtype is enriched for triple negative (TN) tumors (ER-/PR-/HER2-). The HER2E subtype captures some but not all HER2+ tumors. An alternative classification scheme based on copy number and transcriptional profiling are the 10 integrative clusters (IntClusts), with distinct copy number profiles and genomic drivers, and with HER2+ tumors almost fully captured by IntClust5 [2–4]. Breast cancer subtypes, defined transcriptionally, by copy number or IHC/ISH, drive very distinct treatment options: hormone therapy for ER+ tumors, chemotherapy for TN tumors, and HER2-targeted therapy for HER2+ tumors. A fourth protein marker, androgen receptor (AR), is a member of the steroid receptor family and is expressed in 60–80% of breast tumors at levels comparable to prostate cancer [5]. Recent reports have proposed “molecular apocrine” as an additional subtype of breast cancer, characterized by increased androgen signaling and apocrine differentiation [5–7].

Across cancers, classification of tumors by broad expression profiling is increasingly used for drug development and individual treatment decisions. PAM50, for example, is available in both Europe and the USA via the Prosigna™ assay (www.prosigna.com). In retrospective analysis of breast cancer trials, HER2E tumors with HER2 amplification were observed to benefit most from anti-HER2 agents, luminal A and B tumors with HER2 amplification were likely to benefit from anti-HER2 targeted therapy, and basal-like tumors with HER2 amplification benefited least [8–11]. It is speculated that intrinsic subtypes will not replace clinical HER2 assessment, but may influence treatment for the subset of basal-like HER2A tumors [11]. Wider use of these subtypes in the clinic is thus expected [12]. Additionally, cancers are highly diverse, and transcriptional profiling can often omit important aspects in the desire to provide simple, distinct subtypes. These emphasize the need to better understand the underlying drivers and biology of HER2-amplified (HER2A) and HER2E breast tumors.

We explored the nature of HER2A breast cancer using genomic profiles of 3155 breast tumors from The Cancer Genome Atlas (TCGA) [13], the Metabric consortium [2], and the USO1062 clinical trial [14] (Additional file 1). We confirm that HER2 amplification is seen in all PAM50 subtypes, with more than half outside of the HER2E subtype [15]. Only half of HER2E tumors are HER2A. A careful examination of the transcriptional HER2E subtype revealed that these tumors are enriched for ER-negative, yet AR-driven tumors. There is a therapeutic opportunity to treat AR-driven tumors with anti-androgen agents, or combine such agents with anti-HER2 therapy when HER2 amplified, similarly to some routine treatment of ER+/HER2+ tumors with both HER2-targeted agents and anti-estrogens. Beyond breast cancer, anti-HER2 agents are only approved to date for gastric and gastro-esophageal junction cancers. Focal amplification of the HER2 locus in other cancers suggests that more patients may benefit from HER2-targeted treatments. The observed differences in the impact of HER2A on HER2 transcript and protein across cancers may enable prediction of therapeutic efficacy.

## Methods

### Data

Genomic data are from TCGA [13], Metabric [2], and the USO1062 phase III trial [14]. A flow chart and metadata for these three cohorts are available in Additional file 1.

#### TCGA

RNA sequencing (RNAseq) data are from NCI's Genomic Data Commons (https://gdc.cancer.gov) and were analyzed with HTSeqGenie [16]. Gene expression was quantified as reads per kilobase of exon model per million mapped reads normalized by size factor (nRPKM), defined as the number of reads aligning to a gene in a sample/(total number of uniquely mapped reads for that sample x gene length x size factor). We removed ambiguous genes without a gene symbol, genes of uncertain function (LOC symbols), and low expressed genes (defined as genes with both average nRPKM and 90^th^ percentile nRPKM across all breast tumors < 1). This resulted in RNAseq data on 15464 genes for 994 breast tumors.

Affymetrix SNP6 copy number data are from NCI's Genomic Data Commons (https://gdc.cancer.gov). Data were processed with an internal pipeline based on PICNIC [17], followed by custom quality control. All our analyses use relative (ploidy-corrected) copy number, defined as total copy number relative to the average copy number across the tumor genome (ploidy). We excluded 130 tumors with low-quality data, high background noise, or for which ploidy or normal contamination could not be estimated accurately.

Expression and copy number data were available for 895 tumors. We performed intrinsic subtype classification using the PAM50 predictor as described [1]. Subtype centroids, the training set for the 50-gene classifier, R code to run the classifier, and a guide to the intrinsic subtyping were obtained from https://genome.unc.edu/pubsup/breastGEO. Prior to classification, we reduced platform bias through a training set to test set normalization. We adjusted the entire RNAseq set with a platform correction (gene median centering), obtained from a balanced panel of 200 randomly selected ER+ (IHC) and 200 randomly selected ER- TCGA tumors, to mimic the ER proportion in the PAM50 training set. We also updated the symbols of three genes for which annotation has changed since the original PAM50 publication [1]: *CDCA1* to *NUF2* (Entrez Gene ID 83540), *KNTC2* to *NDC80* (Entrez Gene ID 10403), and *ORC6L* to *ORC6* (Entrez Gene ID 23594).

Of the 895 tumors, 154 were basal-like, 73 HER2-enriched, 423 luminal A, 214 luminal B, and 31 normal-like by PAM50. We excluded the 31 normal-like tumors, based on the hypothesis that normal-like is an artifact of having too few tumor cells and an abundant presence of normal breast and/or stromal cells, supported by a tumor-normal mixing experiment and treatment-induced subtype switching [9, 18]. We also observed that these normal-like samples have lower fractions of tumor nuclei and immune cell infiltration, and higher fractions of normal cells. The average of fractions was used for tumors with multiple slides, obtained from NCI's Genomic Data Commons (https://gdc.cancer.gov).

Exome sequences of tumors and matched normals are from NCI's Genomic Data Commons and were analyzed with HTSeqGenie and the Genome Analysis Toolkit (GATK) for variant calling. Tumor-specific variants were obtained by comparing tumor and matched normal, excluding polymorphic variants from dbSNP version 132 that are not reported in the Catalogue of Somatic Mutations in Cancer (COSMIC) database.

Infinium HumanMethylation450 Beadchip methylation data are from NCI's Genomic Data Commons (https://gdc.cancer.gov). Data were processed with an internal pipeline based on R/BioConductor packages methylumi and methyAnalysis, for quality and color balance assessment, color balance adjustment, background adjustment, normalization and methylation modeling. We used the lumi BioConductor package for the calculation of *M* values, as the log2 ratio of methylated to unmethylated probe intensity.

Reverse phase protein array (RPPA) level-3 data are from NCI's Genomic Data Commons (https://gdc.cancer.gov). The HER2 antibody used for RPPA is the mouse monoclonal MS-325-P1 (Lab Vision) and recognizes the cytoplasmic domain of recombinant human erb-b2/HER2. This antibody has a predominant single band in western blot against cell lines and tumors, lacks nonspecific binding, has similar results to RPPA and western blot, and was therefore certified for use by RPPA [19]. The anti-phospho-HER2 (Tyr1248) antibody used for RPPA is the rabbit polyclonal 06-229 (Upstate, Millipore) and recognizes the major auto-phosphorylation (Tyr1248) site of human HER2 in the cytoplasmic domain. This antibody did not fulfill the validation criteria of showing specificity against tumors stimulated or inhibited to yield phosphorylated or non-phosphorylated forms of HER2 protein [19]. Anti-phospho-HER3 (Tyr1289), anti-phospho-AKT (pan-AKT Ser473), and anti-phospho-p44/42 MAPK (ERK1/2) (Thr202/Tyr204) are all from Cell Signaling, with the latter two validated according to criteria as previously published [19]. The AR antibody is the validated, rabbit monoclonal 1852-1 from Epitomics.

ER IHC was available for 84% of tumors and PR IHC for 83%. HER2 positivity was assessed by TCGA following the American Society of Clinical Oncology (ASCO)/College of American Pathologists (CAP) guidelines for IHC, supplemented with fluorescent in-situ hybridization (FISH) results and/or copy number calls for tumors with equivocal or missing HER2 IHC [13].

#### Metabric

Metabric consists of a discovery cohort of 997 patients with breast cancer and a validation cohort of 995 patients with breast cancer [2]. We accessed data through the European Genome-phenome Archive (EGA). RNA expression array data (Illumina) was collected on 1990 out of 1992 tumors and 144 matched-normal tissues. Data were normalized and the quality assessed with the lumi BioConductor package. Because the Metabric consortium already excluded samples based on their quality control, we did not exclude additional samples. We imputed missing expression values using the *k*-nearest neighbor approach with *k* = 10, using the R package impute. We excluded probes that were detected in less than 1% of tumors, and poor-quality probes (matching repeat sequences, intergenic or intronic regions) or without match to any genomic region or transcript [20]. Retaining only perfect and good probes corresponds to removing lowly expressed probes and probes with high expression caused by non-specific hybridization. Probe measurements were collapsed into a single gene measurement for the ~ 30% of genes represented by multiple probes, by selecting the probe with highest variance across all tumors, using the collapseRows function in the WGCNA package in R. For *ERBB2*, this resulted in the selection of probe ILMN_2352131 (average expression of 10.8 and standard deviation of 1.34 across 1107 tumors compared to probes ILMN_1728761 and ILMN_1717902 with average expression of 6.8–6.9 and standard deviation of 0.11 and 0.07, respectively). Additional removal of genes of uncertain function (LOC symbols) resulted in expression data for 15682 genes in 1990 tumors.

Affymetrix SNP6 copy number data for 1991 tumors were processed with our internal pipeline, in the same way as for TCGA. We excluded 28 tumors with low-quality data and high background noise, and 710 tumors for which ploidy and normal contamination could not be estimated accurately. The importance of ploidy for our ploidy-based definition of HER2 amplification warranted stringent custom quality control. This resulted in high-quality DNA copy number alterations for 1253 breast tumors.

Expression and copy number data were available for 1252 tumors, of which 167 were basal-like, 135 HER2-enriched, 518 luminal A, 287 luminal B, 142 normal-like, and 3 unclassified according to PAM50 calls provided by Metabric [2]. We excluded 145 normal-like or unclassified tumors, resulting in 1107 tumors. Targeted exon sequence data for 173 genes are from Pereira et al. [3].

IHC for ER was performed on 98% of tumors, and IHC for HER2 on 41% of tumors. We defined HER2 positivity as IHC 2+ or 3+. IntClust membership was available for 95% of tumors, of which 202 tumors are ER- by IHC.

#### USO1062

The United States Oncology trial 01062 is an adjuvant study assessing the addition of capecitabine to standard chemotherapy [14]. DNA and RNA were extracted from 1539 formalin-fixed paraffin-embedded breast tumors from patients enrolled onto the trial. Tumors were characterized using an 800-gene expression panel (Nanostring) and/or a 35-gene copy number alteration panel (Fluidigm). Raw expression data were log10-transformed and normalized against included housekeeping genes. PAM50 subtype prediction for Nanostring data was carried out using a random-forest-based classifier derived from an independent training set, using the 50 genes from the public PAM50 classifier. Detailed information can be found in a previous publication [14].

Expression and copy number data were available for 1008 tumors (301 basal-like, 70 HER2-enriched, 490 luminal A, 126 luminal B, and 21 normal-like by PAM50 analysis of Nanostring data). We excluded 21 normal-like tumors, resulting in 987 tumors. ER and PR status for these tumors were assessed with IHC and HER2 status was assessed with IHC and FISH.

### Statistical analyses

We assessed the performance of three HER2A classification schemes: four or more ploidy-corrected copies of HER2, five or more total copies of HER2, and four or more centromere-corrected copies of HER2 (i.e. ratio of HER2 to chromosome 17 centromere copy number, estimated as the average number of copies for 431 genes on 17p) (Additional file 2). Normal mixture modeling (R package mclust) was used to define a bimodal threshold for *HER2* overexpression from RNAseq data (TCGA), Illumina microarray data (Metabric), and Nanostring data (USO1062), and for HER2 and phospho-HER2 protein abundance from RPPA data (TCGA). Voom + limma in R was used for differential gene expression analysis. PAM50 subtype and a measure of chromosomal instability and breakage (CIN) were included in the model when assessing differentially expressed genes between HER2A and non-HER2A breast tumors. CIN is a persistently high rate of loss and gain of chromosomes or chromosomal segments, and can confound differential gene expression when its prevalence is different between HER2A and non-HER2A tumors. We calculated CIN as the total number of segments on the autosomal chromosomes with distinct copy levels. ER expression, PR expression and proliferation score as calculated by the PAM50 algorithm were included in the model when assessing genes differentially expressed between HER2E and non-HER2E breast tumors. Gene set enrichment analysis was done using the camera function in the Bioconductor package limma. Camera is a gene set enrichment test that accounts for correlation between genes that belong to the same gene set [21]. We set the inter-gene correlation value to 0.05, to obtain fewer significant hits that are more biologically interpretable. We assessed the enrichment of the C2 collection from the Molecular Signature Database (MSigDB) [22]. Pathways from the Kyoto Encyclopedia of Genes and Genomes (KEGG) were not considered due to licensing restrictions. Gene sets were filtered by *p* value corrected for multiple testing (false discovery rate (FDR)) [23]. The Cox proportional hazards model was used for survival analysis and to generate hazard ratios (HR), using the survival package in R. We censored data for patients who had not had an event of progressive disease or death at the date of their last tumor assessment. Human reference genome hg19 was used in all analyses.

### AR-ness signature

We built a signature of 14 genes induced by androgen or reflective of active AR signaling (positive signature genes, present in at least two out of three MSigDB C2 gene sets: *Doane breast cancer classes up*, *Doane response to androgen up*, and *Farmer breast cancer cluster 7*), and 31 genes suppressed by androgen (negative signature genes, from gene set *Doane breast cancer classes down*). We did not include genes located on chromosome 17, to not confound the detection of AR-driven tumors with HER2 amplification. The expression of each signature gene was z-score-normalized across the ER- tumors per cohort (i.e. normalized to a mean of 0 and standard deviation of 1). The AR-ness score for an ER- breast tumor was then defined as the average z-scored expression of 14 positive signature genes, minus the average z-scored expression of 31 negative signature genes. Out of 45 signature genes, 42 were available for Metabric. Only nine signature genes were present on the Nanostring platform used for USO1062. These clinical trial tumors were therefore not scored for AR-ness.

### Pan-cancer analyses

We associated HER2 amplification with subtype, for those cancers with well-established subtypes. For gastric, bladder, ovarian and head and neck cancer, we used molecular subtypes derived by TCGA, based on either expression data (bladder, ovary, head and neck) or a diverse panel of molecular data (gastric) [24–27]. For colon cancer, we used the consensus subtypes derived from six independent classification systems [28]. The subtype calls (CMS final network and random forest classifier for non-consensus samples) provided by Guinney and colleagues were used [28].

We defined activating HER2 mutations as those with increased tyrosine kinase activity and cellular signaling, that increase cellular transformation and tumor formation, and/or sensitize tumor cells to HER2-targeted therapies in at least one of the referenced studies [29–31]. This set consists of G309A, S310F, L755S, D769H, D769Y, V777L, V842I, and T862A. Other HER2 mutations include those with no functional effect (e.g. R678Q) or that have not been tested (e.g. L755W).

## Results

### Defining HER2 amplification by genomics

**Fig. 1:**
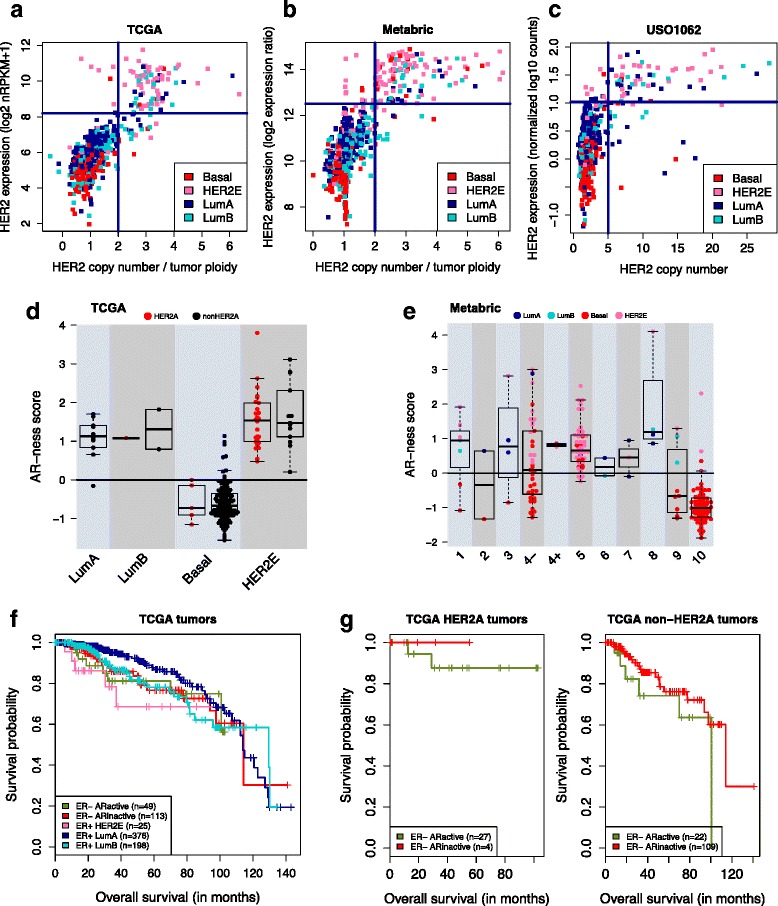
Human epidermal growth factor-2 (HER2) amplification in breast cancer is an event on top of a luminal (Lum), basal or androgen receptor (AR)-driven state. **a**, **b** HER2 expression versus the number of ploidy-corrected HER2 copies. Tumors are colored by prediction analysis of microarray 50 (PAM50) subtype. **a** Among The Cancer Genome Atlas (TCGA) breast tumors, 12.3% (n = 106) are HER2 amplified (HER2A), of which 83 have high HER2 expression (defined as log2(nRPKM + 1) ≥8.2 as per Additional file 3A). *nRPKM* reads per kilobase of exon model per million mapped reads normalized by size factor. **b** Among Metabric tumors, 12% (133/1107) are HER2A, of which 120 overexpress HER2 (defined as log2 expression ratio ≥12.5 as per Additional file 3E). **c** For the USO1062 trial with unavailable tumor ploidy, HER2A was defined as ≥5 copies of HER: 8% (79/987) of USO1062 tumors are HER2A, of which 58 overexpress HER2 (defined as normalized log10 counts ≥1.02 as per Additional file 3G). **d** AR-ness score across PAM50 subtypes for 178 breast tumors from TCGA that are estrogen receptor (ER)- by immunohistochemical staining (IHC). AR-ness score is calculated as the difference in average *z*-scored expression of 14 positive signature genes and average *z*-scored expression of 31 negative signature genes. Tumors are colored by HER2A status. **e** AR-ness score across the integrated clusters (IntClusts) for 202 breast tumors from Metabric that are ER- by IHC. Tumors are colored by PAM50 subtype. IntClust4 is divided into IntClusts 4- and 4+ by ER IHC, as per a previous publication [3]. **f** Kaplan-Meier curve of overall survival (OS) in 761 TCGA tumors with a median follow up of 27 months, divided into five groups based on ER IHC status, PAM50 subtype, and AR activity (positive versus negative AR-ness score). OS was truncated to 12 years of follow up. **g** Left, Kaplan-Meier curve of OS in 31 ER- HER2A tumors from TCGA, divided by AR-ness score. Right, Kaplan-Meier curve of OS in 131 ER- non-HER2A tumors from TCGA, divided by AR-ness score. OS was truncated to 12 years of follow up. *HER2E* HER2-enriched

We defined HER2-amplified (HER2A) tumors as having a ploidy-corrected copy number for HER2 ≥ 4 (i.e. ratio of copy number to ploidy ≥2). Ploidy corrects for the extensive background amplifications seen in breast tumors. This threshold maximized concordance with HER2 over-expression, clinical HER2 status, HER2 protein abundance, and phosphorylated HER2 protein abundance in TCGA and Metabric (see Additional file 2). This definition covers 12.3% (106/864) of TCGA breast tumors (Fig. 1a), and 12% (133/1107) of Metabric tumors (Fig. 1b). Ploidy-corrected HER2A status is 96–98% concordant with HER2 overexpression in the two cohorts, and improves precision by 9–36% compared to two other SNP6-based measures of HER2 amplification (five or more total copies of HER2, or ratio of HER2 to chromosome 17 centromere copy number ≥2) (Additional file 2, Additional file 3A, D-F). Among TCGA HER2A tumors, 78% have elevated HER2 protein levels, compared to 49–70% with alternative measures of HER2A, and 71% have elevated phosphorylated HER2, indicative of activation, in contrast to 43–63% (Additional file 3B-D). Ploidy-corrected HER2A status is also concordant with clinical measures of HER2 copy number and protein levels (HER2+) in both cohorts: 90% of HER2A tumors are clinically HER2+, while only 4.8% of non-HER2A tumors are HER2+ (Additional file 3D, F). Alternative HER2A measures predict only 57–85% of HER2+ tumors (Additional file 3D, F, H). For the USO1062 trial, we considered the 8% (79/987) of tumors with total HER2 copy number ≥5 as HER2A due to unavailability of tumor ploidy (Fig. 1c, Additional file 3H).

### HER2 amplification is a discrete event found in all breast cancer subtypes

Concordance between HER2A and the PAM50 HER2E subtype was remarkably weak: only 47% of HER2A tumors are HER2E while 18% are luminal A, 24% luminal B, and 11% basal-like across the three cohorts combined (Table 1, Additional file 3D, F, H). This genomically confirms the prior observation that half of clinical HER2+ tumors fall in the HER2E subtype, while the rest are observed predominantly in the luminal subtypes [13]. The non-HER2E tumors that are HER2A are very clearly classified as luminal or basal-like by PAM50 despite HER2 amplification, with their PAM50 subtype scores comparable to those of non-amplified tumors (Additional file 4A-B). HER2A is thus a genomic event found across all PAM50 subtypes, while the HER2E subtype, of which 46% are non-HER2A tumors, may be driven by additional factors. Given this strong discrepancy, we set out to understand the genomic correlates of HER2 amplification across all subtypes, and to understand the additional factors beyond HER2A driving the HER2E subtype.

**Table 1:** HER2 is amplified in all PAM50 subtypes, and enriched in HER2E

Subtype	TCGA (n = 106)	Metabric (n = 133)	USO1062 (n = 79)
HER2E	48 (45%)	71 (53%)	30 (38%)
Luminal A	19 (18%)	14 (11%)	26 (33%)
Luminal B	32 (30%)	27 (20%)	17 (21%)
Basal-like	7 (7%)	21 (16%)	6 (8%)

Number and percentage of human epidermal growth factor receptor 2 amplified (HER2A) tumors across the prediction analysis of microarray 50 (PAM50) subtypes in The Cancer Genome Atlas (TCGA), Metabric and the USO1062 clinical trial. *HER2E* HER2-enriched

We assessed the genomic correlates of HER2 amplification by comparing amplified and non-amplified tumors across all subtypes. We focused on mutations and copy number alterations in 43 genes previously shown by TCGA to be frequently altered in breast cancer [13] (Additional file 5). The mutation and copy number profile of HER2A tumors largely reflect those of the underlying subtype, rather than those driven by HER2 amplification (Additional file 4C). Only three mutations and copy number alterations have a significant association with HER2A status in an individual subtype: PIK3CA mutations are more prevalent in basal-like HER2A compared to non-HER2A tumors (40.9% (*n* = 9) vs. 10% (24)); GATA binding protein 3 (GATA3) mutations are more prevalent in HER2E tumors without HER2 amplification (1.8% (2) vs. 13.3% (11)); and BRCA1 deletions are more prevalent in HER2E tumors with HER2 amplification (21.8% (n = 26) vs. 4.5% (4)) (Additional file 4C). HER2A also shows no evidence of being a transcriptional subtype. Only 43 protein-coding genes are differentially expressed between HER2A and non-HER2A breast tumors, when accounting for PAM50 subtype and chromosomal instability (Additional file 4D, Additional file 6A). Twenty-nine of these are neighbors of HER2 on 17q12-21 and can be explained by co-amplification. Expression of genes outside of this region is moderately impacted (<3 times altered in any cohort), with the exception of two secretoglobins: mammaglobin A (*SCGB2A2*) and lipophilin B (*SCGB1D2*). These are chromosomal neighbors on 11q13, form a covalent complex [32], and are 4–8 times more highly expressed in HER2A compared to non-HER2A TCGA tumors (Additional file 4E). Other reported HER2 target genes did not validate when correcting for subtype and chromosomal instability [33, 34]. HER2 amplification thus shows minor association with transcriptional changes outside 17q12-21, consistent with previous findings based on clinical HER2+ status [7, 15]. Taken together, we found that HER2 amplification is a discrete event on top of a luminal or basal transcriptional and mutational state.

### What is HER2E if it is not defined by HER2 amplification?

While HER2E tumors are believed to be HER2-driven, only half of HER2E tumors are HER2A. This brought into question whether HER2E is a consistent subtype. We set out to better understand the molecular composition of the HER2E subtype, other than increased transcription of HER2 and GRB7, the two amplicon genes on the PAM50 panel. We performed gene set enrichment analysis between HER2E and non-HER2E TCGA tumors, accounting for ER expression, PR expression and PAM50 proliferation score, and omitting genes on chromosome 17 to reduce interference with HER2 amplification (Additional file 7A-B). The most significant gene set enriched in HER2E tumors (*Doane breast cancer classes up*; Additional file 8A) is composed of genes upregulated in a subset of ER-/PR- tumors but that, paradoxically, are direct targets of ER, responsive to estrogen, and/or typically expressed in ER+ breast cancer [35]. Genes downregulated in those ER-/PR- tumors are concordantly lower expressed in the HER2E tumors (hit #7; Additional file 8A).

The answer to this puzzling expression pattern may be in androgen receptor (AR), another steroid hormone receptor that is highly expressed in some breast tumors, has overlapping target genes with ER, and is on average eight times more highly expressed in HER2E compared to non-HER2E tumors (Additional file 7A). Indeed, MDA-MB-453, a TNBC cell line that expresses those paradoxical genes and lacks typical basal-like cytokeratins, was shown to respond to androgen in an AR-dependent and ER-independent manner, and its expression profile is, at least in part, AR-induced [35]. This was confirmed independently in vivo for AR antagonist bicalutamide [36]. Additional evidence indicative of androgen signaling in HER2E tumors is the enrichment of gene set *Farmer breast cancer cluster 7* (hit #6; Additional file 8A). These genes were found to be highly expressed in breast tumors considered molecular apocrine, based on their active AR signaling (i.e., expression of genes induced by androgen in LNCaP prostate cancer cells), weak ER signaling, and morphological hallmarks of apocrine tumors such as abundant eosinophilic cytoplasm and prominent nucleoli [6]. Beyond these sets, three additional gene sets in the top 10 (hits #3, 4 and 9; Additional file 8A) indicate that HER2E tumors display a transcriptional profile that is more similar to ER+ breast tumors than to the basal subtype, despite the shared ER- status [6, 35, 37]. This is supported by expression of luminal cytokeratins (KRT7, KRT8, KRT18), luminal markers FOXA1 and XBP1, and lack of basal-like cytokeratins such as KRT5, KRT6A, and KRT81 (Additional file 7A). Furthermore, HER2E tumors not only selectively express genes that were previously observed in ER-/AR+ or molecular apocrine breast tumors, but also genes induced by androgen [35] (Additional file 7B). The minimal gene overlap between these sets increases our confidence that they independently support overlap between HER2E and androgen signaling (Additional file 8B). These gene set enrichment results suggest that AR regulates the transcriptional program of HER2E tumors.

### AR-ness signature identifies ER- tumors with androgen-driven transcriptional program

Other subtypes besides HER2E may also contain AR-driven tumors. We therefore derived a signature of AR-ness that is agnostic to subtype. We selected 14 genes included in at least two of three gene sets reflective of active AR signaling (hits #1, 6 and 69; Additional file 7B), and 31 AR-repressed genes (hit #7). This resulted in a 45-gene AR-ness signature (Additional file 7C). Because AR is co-expressed with ER in up to 90% of ER+ breast cancer [38] and AR can recapitulate the ER-mediated transcriptional program seen in luminal breast cancers [39], we applied the AR-ness signature to tumors that are ER- by IHC, to identify ER- tumors with apocrine features, active AR signaling, and/or expressing androgen-induced genes. As shown in Fig. 1d and Additional file 8C, basal-like tumors score in general low for the AR-ness signature, that is, androgen-induced genes are on average lower expressed than genes reflective of inactive AR signaling (TCGA: 116/126, 92%; Metabric: 100/121, 83%). HER2E ER- tumors on the other hand score high for AR-ness (TCGA: 39/39, 100%; Metabric: 62/67, 93%). In the context of the IntClusts, most of the AR-driven Metabric tumors reside in IntClust4-, which consists of ER- tumors with favorable outcome, and in IntClust5, which captures most of HER2-amplified cancers regardless of ER status [4] (Fig. 1e). ER- TCGA tumors with a positive AR-ness score have concordant higher abundance of AR protein levels (*t* test, *p* = 3e-7; Additional file 8D). Across subtypes, we found that 34% (61/178) of ER- TCGA tumors and 48% (98/205) of ER- Metabric tumors have a positive AR-ness score. These prevalence rates are similar to those observed by others [6, 35, 36].

Around half of AR-driven ER- tumors are HER2A (TCGA 46%, Metabric 58%). Subdivision by subtype revealed that two thirds of HER2E tumors are HER2A, and AR-ness score is consistent across HER2A and non-HER2A tumors of this subtype, showcasing that HER2E is a consistent subtype independent of HER2A status (Fig. 1d, Additional file 8C). Results for basal-like tumors differed by cohort. In TCGA with only 10 AR-driven basal-like tumors, AR-ness scores did not differ by HER2A status (Fig. 1d). In Metabric, AR-ness scores were significantly higher in HER2A basal-like tumors (*t* test, *p* = 3.2e-5), and concordantly the 21 AR-driven basal-like tumors were enriched for HER2A (52% vs. 3% of AR-inactive tumors; Fisher’s exact test, *p* = 8e-8) (Additional file 8C). These findings suggest crosstalk between HER2 amplification and AR activity in certain contexts.

### Treatment alternatives for AR-driven breast tumors

The finding that ER- and/or HER2A tumors are frequently AR-driven suggests that those tumors may benefit from treatment regimens including AR antagonists. TN tumors with a luminal AR-driven (LAR) profile, defined as expressing AR and downstream AR targets and co-activators [36], have been shown to benefit less from standard neoadjuvant chemotherapy compared to other TN breast tumors [40]. Eight out of nine LAR-specific genes are significantly higher expressed in HER2E tumors, independent of HER2A status (Additional file 7A). Our signature thus not only identifies TN LAR tumors, covering basal-like, HER2E and IntClust4-, but also HER2A tumors. While AR antagonists may be beneficial for this subset of TN and HER2A tumors, this may not be the case for luminal tumors. The luminal A and B tumors with high AR-ness score but that were ER- by IHC had ESR1 expression levels comparable to ER+ luminal tumors, suggesting that endocrine therapy could suffice.

To support this hypothesis, we assessed prognosis in TCGA and Metabric. In TCGA, HER2E ER+ tumors were associated with the worst and luminal A tumors with the best overall survival, and this was the case in both HER2A and non-HER2A tumors (multivariate Cox proportional hazards model with HER2A status and subtype based on PAM50, ER IHC and AR-ness score; hazard ratio (HR) ER+ luminal A vs. ER+ HER2E, 0.301, 95% CI 0.109–0.833) (Fig. 1f). In Metabric, where women were enrolled before the general availability of HER2-targeted agents, both HER2E and basal tumors were associated with significantly worse survival than luminal A tumors, independent of AR activity and HER2A status (Additional file 8E).

Specifically for ER- tumors, there is a trend in TCGA towards worse survival in patients with ER- tumors with active AR signaling compared to ER- tumors with inactive AR signaling in both HER2A and non-HER2A tumors (multivariate HR 1.853, 95% CI 0.745–4.608) (Fig. 1g). Though not conclusive, this suggests that patients with ER- tumors with active AR signaling do worse on chemotherapy, the current standard of care for TNBC, and could potentially benefit from alternative treatment options including AR antagonists. AR activity was not prognostic in Metabric (Additional file 8F), possibly impacted by lack of exposure of HER2A Metabric tumors to HER2-targeted agents [4].

### Do HER2A tumors share co-operating drivers?

**Fig. 2:**
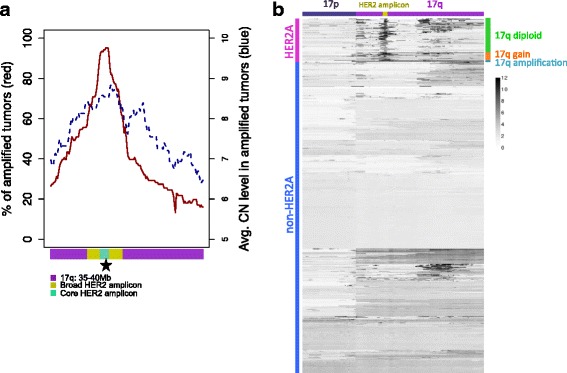
Characterization of the human epidermal growth factor receptor 2 (HER2) amplicon and HER2 co-amplification in breast cancer. **a** Percentage of 106 HER2 amplified (HER2A) tumors from The Cancer Genome Atlas (TCGA) with gene amplification (solid red line, left axis), and average copy number (CN) level in HER2A tumors with gene amplification (indicated in dashed blue line, right axis), for genes on chromosome 17 from 35 Mb to 40 Mb (ordered by genomic location). Shown at the bottom are core HER2 amplicon (10) and broad HER2 amplicon (37) genes. The HER2 locus is starred. **b** Copy number of genes on chromosome 17 is shown for HER2A (top) and non-HER2A (bottom) TCGA tumors. Three distinct groups of HER2A tumors are labeled on the right: tumors with 17q arm-level amplification, defined as 5 or more copies for at least 80% of genes (cyan); tumors with 17q gain (copy number between 2.5 and 5 for 80% or more genes) (orange); and tumors that are mainly 17q diploid with copy number <2.5 for the majority of 17q genes (green). Chromosome 17 annotation is indicated on top. Regions 34.4–34.6 Mb and 44.1–44.8 Mb with germline micro-deletions or micro-gains were removed for visual purposes (see Additional file 9F-G)

Next, we explored the extent of amplification near HER2 and its impact on gene expression, to identify putative HER2-cooperating oncogenic drivers. HER2A breast tumors are more chromosomally instable than non-HER2A tumors (Additional file 9A), which in the past led to the hypothesis that HER2 amplification drives the selection of additional copy number aberrations [41, 42]. The HER2 amplicon has no conserved breakpoints, but does have a minimal core of genes amplified in most tumors (Fig. 2a, Additional file 9B-C). Ten genes within the core HER2 amplicon, spanning 237 kb, are amplified to five or more total copies in at least 92% of HER2A TCGA tumors. A broad HER2 amplicon with genes amplified in at least 60% of HER2A TCGA tumors covers 1.14 Mb and 37 genes from *LRRC37A11P* to *CASC3*. We confirmed the HER2 amplicon boundaries in the Metabric cohort (Additional file 9C), concordant with previous publications [42, 43]. Four regions on chromosome 17 outside of the broad HER2 amplicon are significantly co-amplified with HER2 in the combined TCGA and Metabric cohorts when taking chromosomal instability into account (Fig. 2b, Table 2). One of these regions is adjacent to the HER2 amplicon. Genes in the other three regions, including 11 cancer genes (as reported [44, 45]), are amplified in 3.4% to 27.6% of HER2A tumors and in a maximum 5.6% of non-HER2A tumors (Additional file 10). Taken together, chromosome 17 and in particular the HER2 amplicon are likely to include HER2-cooperating drivers such as *GRB7*, *STARD3*, *MIEN1*, and *LASP1* [43, 46, 47]. Most tumors have focal HER2A amplification on top of a largely diploid 17q, but 19% of TCGA HER2A tumors have arm-level gain, and 4% are defined as HER2A due to 17q amplification without further focal HER2 amplification (Fig. 2b, Additional file 9D).

**Table 2:** HER2 co-amplification in breast cancer

Chr	# Genes	Band	Start - end gene	Median amplified percentage in HER2A (IQR)	Median amplified percentage in non-HER2A (IQR)
3	1	3p25.1	MRPS25	4.2	1.1
3	5	3p24.1	KCNH8 - PP2D1	2.9 (0)	0.52 (0)
6	12	6q21	AIM1 - SNX3	7.5 (0.8)	2.6 (0.5)
10	10	10q22.3	ZMIZ1 - SFTPA1	6.5 (0.8)	1.7 (0.05)
11	34	11q12.3	NXF1 - PYGM	2.5 (0.4)	0.4 (0.06)
13	5	13q12.2-q12.3	FAM123A - SLC46A3	5.0 (0.4)	1.4 (0.3)
14	29	14q11.2	OR4K15 - RNASE1	4.2 (0.8)	0.7 (0.06)
14	15	14q11.2	OR5AU1 - OR4E2	3.4 (0.2)	0.6 (0.06)
17	55	17p11.2	LRRC48 - MTRNR2L1	5.0 (1.9)	1.5 (0.8)
17	419	17q11.1-q21.3	WSB1 - PPY	14.6 (12.3)	0.8 (0.7)
17	296	17q21.3-q24.3	NSF - MAP2K6	16.3 (7.3)	4.3 (1.7)
17	45	17q25.1	RPL38 - SAP30BP	9.2 (2.1)	2.9 (0.3)
20	3	20q13.2	ZFP64 - ZNF217	12.6 (0.8)	6.4 (0.6)
22	1	22q11.1	GAB4	2.5	0.3

Genes that are significantly co-amplified with human epidermal growth factor receptor 2 (HER2) in The Cancer Genome Atlas (TCGA) and Metabric cohorts combined (Fisher’s exact false discovery rate (FDR) *p* value <0.05) (see Additional file 9E, Additional file 10). Median percentage of amplified genes (interquartile range, IQR) is shown per co-amplified region, in HER2 amplified (HER2A) and non-HER2A tumors

Outside of chromosome 17, we identified 10 significantly co-amplified regions containing 116 genes (Table 2, Additional file 9E, Additional file 10). These genes, though often co-amplified with HER2, are not more highly expressed in HER2A than non-HER2A tumors (Additional file 6A), and only one of these is a known cancer gene, ubiquitin ligase *CCNB1IP1*. Genes *SCGB1D2* and *SCGB2A2* that are differentially expressed by HER2A status (FC >4) are not significantly co-amplified with HER2. We thus find no evidence for co-operating copy number drivers with HER2 outside chromosome 17.

### The nature and role of HER2A in other cancers

HER2 amplification is prevalent in several other cancers. We found 1.8% of all primary non-breast TCGA tumors to be HER2A (Table 3). An even higher rate of 3.4% was seen in another cohort of ~ 7300 solid tumors, encompassing primary, locally recurrent, and metastatic tumors [48]. To date, anti-HER2 therapies are indicated for the treatment of breast and metastatic gastric cancer [49]. Based on TCGA HER2A prevalence, these two cancers are estimated to annually account for ~ 31000 new cases in the USA (Table 3) [50]. Other cancers may account for another ~ 14750 new HER2A cases. We explored the genomics of these HER2A tumors to better understand similarities and differences to HER2A breast cancer, and potential for therapeutic intervention.

**Table 3:** Estimated HER2A patient population size based on HER2A prevalence in 5391 non-breast tumors from TCGA

Cancer	HER2A percentage	Estimated number of new cases in USA, 2015	Estimated number of new HER2A cases
Breast carcinoma	12.3% (106/864)	234190	28805
Stomach adenocarcinoma	8.9% (24/271)	24590	2189
Bladder carcinoma	4.2% (7/168)	74000	3083
Cervical squamous cell carcinoma and uterine carcinosarcoma	4.1% (7/172)	12900	529
Uterine corpus endometrioid carcinoma	3.4% (12/355)	54870	1866
Ovarian serous cystadenocarcinoma	2.7% (11/401)	21290	575
Colon adenocarcinoma	2.5% (5/203)	93090	2327
Lung adenocarcinoma and squamous cell carcinoma	2.4% (21/884)	221200	5309
Head and neck squamous cell carcinoma	2.3% (9/384)	45780	1053
Kidney renal papillary cell carcinoma	1.3% (2/151)	N/A	N/A
Total across non-BC with HER2 amplification (11 cancers)	3.3% (98/2989)		16931
Total across all non-BC (23 cancers)	1.8% (98/5391)		

Estimate of the number of new, annual human epidermal growth factor receptor 2 (HER2) amplified (HER2A) cancer cases in the USA, based on cancer figures from the American Cancer Society [50] and prevalence of HER2A in The Cancer Genome Atlas (TCGA). *BC* breast cancer, *N/A* not available

**Fig. 3:**
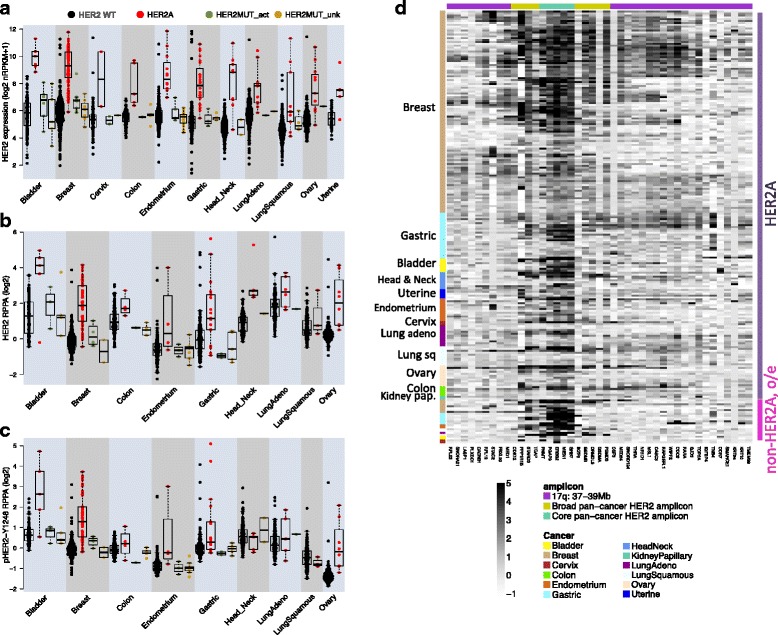
The nature and role of human epidermal growth factor receptor 2 (HER2) amplified (HER2A) in other cancers. **a**–**c** Tumors are grouped by HER2 amplification and mutation status. Tumors without HER2 amplification or mutation are shown in black, HER2A tumors (regardless of HER2 mutation) in red, unamplified tumors with an activating HER2 mutation (HER2MUT act) in green, and unamplified tumors with an untested or functionally inactive HER2 mutation (HER2MUT unk) in gold. **a** HER2 expression is consistently higher in HER2A tumors than in tumors without HER2 alteration, across all cancers (linear model with HER2 status and cancer, *p* = 0). Non-HER2A tumors with an activating or non-functional HER2 mutation have similar HER2 expression levels to unaltered tumors. **b** HER2 protein levels are higher in HER2A compared to unaltered tumors (*p* = 1e-104), though insignificant in lung squamous cell carcinoma (*p* = 0.08). HER2 protein levels in tumors with activating or non-functional HER2 mutations are similar to unaltered tumors. **c** Phospho-HER2 (Tyr1248) levels are significantly higher in HER2A bladder (*p* = 8e-12), breast (*p* = 3e-53), gastric (*p* = 1e-10), ovarian (*p* = 8e-20), and endometrial (*p* = 1e-8) tumors compared to unaltered tumors. pHER2 levels in tumors with activating or non-functional HER2 mutations are similar to unaltered tumors. **d** Expression of genes in a 2-Mb region around HER2, in a panel of HER2A tumors (top) and non-HER2A tumors with HER2 overexpression (o/e) (bottom). Expression is normalized per cancer to the median expression of each gene in tumors with 2 or fewer HER2 copies. The 2.5% lowest and highest values are saturated for better contrast. Genes are colored by core and broad pan-cancer HER2 amplicon

HER2-targeted therapy may be an opportunity for HER2A bladder, endometrial, and ovarian cancer. HER2 expression, protein, and phospho-protein levels are higher in HER2A compared to non-HER2A tumors of these types in addition to gastric cancer (Fig. 3a-c). HER2 transcript levels are also higher in HER2A colon, lung, and head and neck carcinoma, but this does not translate to increased HER2 protein and/or pHER2 levels. Levels of downstream phosphorylation markers for ERBB3, pan-AKT, and ERK1/2 are not affected by HER2 amplification (Additional file 11A-C). HER2 may also carry mutations in the kinase and extracellular domains, some implicated in tumorigenesis [29, 51]. Three percent of breast tumors in both the TCGA and Metabric cohort carry an HER2 protein-altering mutation (Additional file 11D). Prevalence in non-breast cancers varies from 0.4% (ovary) to 8.6% (bladder) (Additional file 11D), consistent with previous studies [48, 51]. These mutations are mutually exclusive with HER2 amplification in 92% of mutated tumors (Additional file 11D). In the absence of HER2 amplification, HER2 mutations do not increase HER2 or pHER2 levels (Fig. 3b-c).

HER2A tumors in these cancers share certain similarities with breast cancer. We observed the same pattern in 17q arm-level gain and amplification (Additional file 12A): 51% (n = 50) of non-breast HER2A tumors have focal HER2 amplification on top of a largely diploid 17q, 48% (n = 47) show additional arm-level gain, and one uterine tumor only has arm-level amplification. As in breast, HER2 amplification is found in multiple transcriptional subtypes of bladder, colon, ovarian, and head and neck cancer (Additional file 12B). Non-breast HER2A tumors show focality of HER2 and closest neighboring genes. The core HER2 amplicon in non-breast cancers (amplified in at least 80% of HER2A) covers 79 kb and six genes, from *PNMT* to *GRB7* (Additional file 12C). The broad pan-cancer HER2 amplicon shared by at least 60% of HER2A tumors spans 532 kb, contains 13 additional genes from *CDK12* to *PSMD3*, and is narrower compared to breast. Genes in the broad amplicon have on average 0.85 fewer copies in non-breast compared to breast HER2A tumors (Fig. 2a, Additional file 12C). Expression levels of amplicon genes also vary by cancer (Fig. 3d). Relative expression of core amplicon genes normalized to levels in HER2-diploid tumors are similar in HER2A breast, bladder, head and neck, endometrial, cervical, ovarian, and colon cancers, and are significantly lower in HER2A gastric, lung, kidney, and uterine tumors (one-sided *t* test in comparison to HER2A breast, FDR *p* = 0.013, 0.001, 0.003, and 0.025, respectively). Relative expression levels of broad amplicon genes outside of the core are only lower in lung adenocarcinoma and kidney HER2A tumors in comparison to breast (both FDR *p* = 0.021) (Fig. 3d).

HER2 amplification does not induce transcriptional changes outside of the amplicon in a coherent pan-cancer manner. Only 14 protein-coding genes, all located on 17q12-21, consistently distinguish pan-cancer HER2A from non-HER2A tumors (Additional file 6B). Concordantly, of the 43 genes differentially expressed by HER2A status in breast cancer (Additional file 6A), only genes on 17q12-21 show consistent higher expression in HER2A non-breast tumors (Additional file 12D). The non-amplicon SCGB targets are not consistently higher in HER2A tumors (Additional file 12E).

We also discovered a set of tumors that express HER2 at levels found in HER2A tumors, but which are not amplified (31 breast tumors, Fig. 1a-c; and 16 other TCGA tumors, Fig. 3d, bottom). These tumors, other than cervix and bladder, consistently upregulate the closest HER2-neighboring genes *PGAP3*, *MIEN1*, and *GRB7*, at levels higher than observed in HER2A tumors (Additional file 13A–H). More distal HER2 neighbors *MED1*, *CDK12*, *NR1D1*, and *TOP2A* are not overexpressed. Overexpression of HER2 and closest HER2-neighbors in non-HER2A tumors is not driven by amplification of those genes (Additional file 13I). This suggests that there is a regional control of gene expression. We assessed epigenetic changes and found that high expression of HER2 and closest HER2-neighbors in those tumors is associated with CpG hypomethylation in the bodies or maximum 2 kb upstream of these genes (Additional file 13J-M). The two non-HER2A bladder tumors that overexpress HER2 but lack transcriptional regional control (Additional file 13E) concordantly did not undergo reduced methylation (Additional file 13M). HER2-neighboring genes have been suggested to contribute to HER2A cancer [43, 46]. The co-expression supports the model that co-amplified genes near HER2 contribute to oncogenesis. This small pan-cancer population of non-HER2A tumors with substantial overexpression of HER2 and neighboring genes may also benefit from HER2-targeted treatment.

## Discussion

Tumors from the same tissue may have very diverse mechanisms, genomics, prognosis, and treatment needs. Broad subtypes can therefore oversimplify a complex mosaic of tumor mechanisms and cells of origin, and finer grade classification will be needed for more personalized medicine. In breast cancer, HER2 amplification had been confounded with the transcriptional subtype HER2E. We find that HER2 amplification is a driver event rather than a subtype, is found in all subtypes, and its strong enrichment in the HER2E subtype had masked the nature of this subtype. A careful examination of the transcriptional HER2E subtype revealed that HER2E tumors are hormonally driven, either by ER in ER+ HER2E tumors, or by AR in ER- HER2E tumors. More broadly, we can conclude that ER- tumors that score positive for AR-ness are enriched among HER2E, IntClust4-, IntClust5 and HER2A tumors, but are not fully captured with either subtype. This suggests that a diagnostic signature such as the AR-ness score may be beneficial for a more accurate classification of ER- tumors that would benefit from AR antagonists. Retrospective validation in appropriate trials is required. Of note, genes that were previously shown to change in response to androgens R-1881 in LNCaP cells or dihydrotestosterone in ovarian cells distinguished HER2E from non-HER2E tumors to a lesser extent or not at all (Additional file 7B), indicating that signatures of AR activity may be tissue-specific.

Half of AR-driven ER- tumors are HER2A. The co-occurrence of AR signaling and HER2 amplification, together with previously observed functional crosstalk between the AR and HER2 signaling pathways [52], point towards a therapeutic opportunity to combine AR inhibition with anti-HER2 therapy for better neutralization of oncogenic HER2 in AR-driven breast tumors. In prostate cancer, HER2 signaling has been shown to stabilize AR protein and optimize binding of AR to promoters of androgen-regulated genes, and HER2 pathway inhibition reduces AR transcriptional activity [53]. In molecular apocrine breast cancer, AR has been shown to directly induce HER2 expression, and AR is upregulated by HER2-stimulated ERK activity. Furthermore, combined inhibition of AR and downstream signaling of HER2 synergistically blocks proliferation, and excessive AR activation is needed to trigger the oncogenic potential of HER2 in HER2-amplified, molecular apocrine tumors [52]. These preclinical data are consistent with the high rate of HER2 amplification among AR-driven ER- tumors, suggesting that AR is an insufficient driver in the absence of ER that requires additional tumorigenic events such as HER2 amplification.

AR antagonists bicalutamide (AstraZeneca) [54] and enzalutamide (Medivation) [55] are approved for metastatic prostate cancer, and are being tested in breast cancer. Bicalutamide reached a 19% clinical benefit rate in a phase 2 trial in ER-/PR- breast tumors expressing AR [56]. Enzalutamide, a more potent AR inhibitor, achieved single-agent activity in 20–40% of advanced TN breast tumors expressing AR [57]. These response rates are comparable to the prevalence of AR-driven ER- tumors as detected with our AR-ness signature. For HER2A tumors, a phase 2 trial was initiated in 2014 to evaluate the safety and efficacy of combining enzalutamide and trastuzumab in patients with metastatic or locally advanced breast cancer that are HER2A and molecular apocrine, whose disease previously progressed on trastuzumab (NCT02091960).

Focal amplification of the HER2 locus in non-breast cancers suggests that HER2 is a targetable driver in subsets of other cancers, and indeed, trastuzumab has been approved for gastric and gastro-esophageal junction cancers. Our analysis indicates that bladder, endometrial, and ovarian cancers have the most potential to benefit from HER2-targeted treatment, based on high HER2 levels, but other cancer types may also respond. For instance, the NSCLC cell line Calu-3 is HER2+ by IHC and western blot, and responds to T-DM1 and pertuzumab in vitro and in vivo [58]. Trastuzumab and pertuzumab are being tested in advanced solid tumors with HER2 overexpression outside of the approved indications [59] (NCT02091141).

Besides HER2 amplification, clinical trials are ongoing to evaluate the efficacy of anti-HER2 agents in HER2 mutant but not amplified tumors (NCT01670877 in breast cancer and NCT01827267 in non-small cell lung cancer, both with the irreversible tyrosine kinase inhibitor neratinib). We showed in a large pan-cancer cohort that non-HER2A tumors with either an activating or non-functional HER2 mutation do not have elevated levels of phosphorylated HER2. This supports recent in vitro and in vivo work by Zabransky and colleagues suggesting that HER2 missense mutations, when present alone, are insufficient drivers of growth and metastasis [60].

## Conclusions

We explored the genomics of HER2 amplification in 3155 breast tumors across three cohorts. While HER2 amplification is traditionally associated with the HER2E transcriptional subtype, we observed that the two are substantially distinct. We found HER2 amplification in other intrinsic subtypes, and used this to categorize the landscape of HER2 amplification, independent of subtype. We also found that HER2E has a distinctive transcriptional landscape independent of HER2A, and that this likely reflects AR signaling as a possible replacement for ER-driven signaling. We propose that the HER2E category be recognized as AR-related, and showed that HER2 amplification is an oncogenic driver, found in all subtypes, rather than a marker of any intrinsic subtype. Beyond breast cancer, HER2 amplification is consistently a pan-cancer event that builds on top of transcriptional subtypes, and we propose candidate cancers for HER2-targeted treatment.

## Additional files

Additional file 1: Flow chart summarizing sample size and available data for the TCGA, Metabric and USO1062 cohort; Clinical metadata, subtype information, HER2A status, AR-ness score, HER2 copy number, and expression levels of *ERBB2*, *ER*, *PR* and *AR* for the tumors in these three cohorts. (XLSX 669 kb)

Additional file 2: Robust development of the CN/ploidy definition, and comparison with two alternative HER2A measures. (PDF 115 kb)

Additional file 3: Definition of HER2-amplified breast cancer. (A) HER2 overexpression in 864 TCGA tumors is defined as log2 (nRPKM + 1) ≥8.2 (normal mixture modeling). (B) Abundant HER2 protein in 367 TCGA tumors is defined as log2 (RPPA) ≥0.92. (C) Abundant phosphorylated HER2 (Tyr1248) is defined as log2 (RPPA) ≥0.605. (D) Concordance of three HER2A classification schemes with HER2 expression, HER2 protein, phosphorylated HER2, clinical HER2 status and PAM50 subtype in TCGA (Additional file 2). In each case, concordance to HER2 gene expression drops with the alternative measures, from 96.5% to 94.7% for total copies (McNemar test *p* = 0.002), and to 86.1% for centromere-corrected copies (*p* = 2e-19). Concordance with HER2 protein levels drops from 94.1% to 92.8% (*p* = 0.18) and 86.6% (*p* = 7e-6), respectively. Concordance with pHER2 protein levels drops from 93.6% to 91.7% (*p* = 0.05) and 85% (*p* = 2e-7). Concordance with clinical HER2 status drops from 94.9% to 94.0% (p = 0.11) and 86.7% (*p* = 2e-10). Ploidy-corrected HER2A captures a larger fraction of the PAM50 HER2E subtype (90.4% concordance for HER2E vs. other subtypes) than either total (89.0%, *p* = 0.025) or centromere-corrected HER2A status (80.0%, *p* = 2e-19). (E) HER2 overexpression in 1107 Metabric tumors is defined as log2 expression ratio ≥12.5. (F) Concordance of three HER2A classification schemes with HER2 expression, clinical HER2 status and PAM50 subtype in Metabric. The concordance to HER2 gene expression drops from 97.7% to 95.1% for total copies (McNemar test *p* = 1e-5), and to 90.9% for centromere-corrected copies (*p* = 5e-17). Concordance with clinical HER2 status drops from 94.1% to 91.5% (*p* = 3e-3) and 90.9% (*p* = 2e-6), respectively. Overlap with PAM50 HER2E drops from 88.6% to 86.8% (*p* = 2e-3) and 82.3% (*p* = 1e-14). (G) HER2 overexpression in 987 USO1062 tumors is defined as normalized log10 counts ≥1.02. (H) Concordance of HER2A status with HER2 overexpression, clinical HER2 status and PAM50 subtype in the USO1062 cohort. (PDF 147 kb)

Additional file 4: HER2 amplification is a discrete event on top of a luminal or basal state, with minor consistent correlation with gene expression. (A) Row-scaled expression of 50 PAM50 genes in 864 TCGA breast tumors, labeled on top by HER2A status and PAM50 subtype. (B) PAM50 scores for TCGA breast tumors categorized by PAM50 subtype and HER2A status. HER2A tumors, in red, are confidently classified as luminal A, luminal B, basal-like, or HER2E, with PAM50 scores within 3.3–4.3% of the PAM50 scores of non-HER2A tumors of the same subtype. (C) Shown are the odds that a genomic alteration in gene A will occur in an HER2A tumor of subtype X compared to the odds of gene A being altered in a non-HER2A tumor of subtype X. Each dot represents the enrichment of alterations in a gene in HER2A compared to non-HER2A tumors of a particular subtype, colored by PAM50, with mutations shown as diamond and copy number alterations as circle. Fisher’s exact *p* values were corrected for multiple testing per PAM50 subtype, and separately for the set of 21 genes for which we assessed mutation status (Additional file 5A-B) and 28 genes for which we assessed copy number alterations (Additional file 5C-D). Significance is defined as FDR *p* value <0.1. Enrichments are based on the combined TCGA and Metabric cohorts. (D) Gene-gene expression correlation in TCGA breast tumors for 43 genes differentially expressed between HER2A and non-HER2A tumors when accounting for PAM50 subtype and chromosomal instability. Two sets of 3 or more highly correlated genes (gene-gene correlation >0.6) are highlighted on the right: 28 genes on 17q12-21 near HER2, and 3 SCGB genes at 11q13 (Additional file 6A). (E) Two genes outside of 17q12-21 are more highly expressed in HER2A than non-HER2A TCGA tumors with FC >4: *SCGB2A2* (FC 7.7) and *SCGB1D2* (FC 4.4). (PDF 390 kb)

Additional file 5: Mutation and copy number profiles of breast tumors. (A) Mutation profile for 23 genes previously shown to be significantly mutated in breast cancer [13] in 679 TCGA breast tumors with exome-seq data. (B) Mutation profile for 21 genes (FOXA1 and MLL3 are not available) in 1039 Metabric tumors with targeted sequence data. (C) Copy number profile for 28 genes previously shown to have subtype-specific copy number alterations [13], in 864 TCGA breast tumors. (D) Copy number profile for 28 genes in 1107 Metabric tumors. PAM50 subtype and HER2A status are provided. (XLSX 417 kb)

Additional file 6: Protein-coding genes associated with HER2 amplification. (A) List of 43 protein-coding genes with subtype-independent expression differences between HER2A and non-HER2A breast tumors, after accounting for PAM50 subtype and differences in chromosomal instability, in the TCGA and Metabric cohort. These genes fulfilled the following criteria in at least one cohort: adjusted *p* value <0.05, and fold change >2 (linear model). (B) List of 14 protein-coding genes with cancer-independent expression differences between HER2A and non-HER2A tumors in a panel of 2838 non-breast TCGA tumors, after accounting for cancer and chromosomal instability. These genes fulfilled the following criteria: adjusted *p* value <0.001, and fold change >2. (XLSX 24 kb)

Additional file 7: Characterization of HER2E tumors and AR-ness signature. (A) Overview of genes differentially expressed between HER2E and non-HER2E TCGA tumors, accounting for ER expression, PR expression, and PAM50 proliferation score, and omitting genes on chromosome 17. (B) Gene set enrichment results for the C2 gene set collection from MSigDB [22] (KEGG gene sets were removed), for the comparison of HER2E versus non-HER2E TCGA tumors. (C) 45-gene AR-ness signature. (XLSX 6449 kb)

Additional file 8: Identification of ER- tumors that are AR-driven. (A) Top 10 C2 gene sets from MSigDB [22] enriched among genes differentially expressed between HER2E and other breast tumors. See Additional file 7B for the full list of C2 gene sets. (B) Overlap in number of genes between the top 10 C2 gene sets and androgen responsive gene sets (from Additional file 7B). (C) AR-ness score across PAM50 subtypes for 205 breast tumors from Metabric that are ER- by IHC. AR-ness score is calculated as the difference in average *z*-scored expression of 14 positive signature genes and average z-scored expression of 31 negative signature genes. Tumors are colored by HER2A status. (D) AR protein levels in 90 ER- TCGA tumors by AR-ness score. (E) Kaplan-Meier curve of overall survival (OS) in 977 Metabric tumors with a median follow up of 7.3 years, divided into 5 groups based on ER IHC status, PAM50 subtype, and AR activity (positive vs. negative AR-ness score). OS was truncated to 17 years of follow up. (F) Left, Kaplan-Meier curve of OS in 57 ER- HER2A tumors from Metabric, divided by AR-ness score. Right, Kaplan-Meier curve of OS in 121 ER- non-HER2A tumors from Metabric, divided by AR-ness score. OS was truncated to 17 years of follow up. (PDF 167 kb)

Additional file 9: Characterization of the HER2 amplicon and HER2 co-amplification in breast cancer. (A) Chromosomal instability, shown as the number of breaks per kb per autosome, is higher in HER2A than non-HER2A TCGA tumors, with one-sided *t* test *p* values per chromosome ranging from 0.37 (chr 5) to 5e-26 (chr 17), and <0.05 for 17/22 autosomes. (B) Copy number levels in 106 HER2A TCGA breast tumors, for genes on chromosome 17 from 35 Mb to 40 Mb (ordered by genomic location). The core HER2 amplicon on top is shown in green, the broad HER2 amplicon in yellow, and genes outside of the broad HER2 amplicon on 17q in magenta. Shown in green on top is correlation between copy number and expression (log2 nRPKM + 1) for each gene across the 864 tumors. (C) HER2 amplicon profile in 133 HER2A Metabric tumors. See Fig. 2a legend for details. (D) Four HER2A TCGA breast tumors show 17q arm-level amplification without additional HER2 focality. Shown are total copy number levels for genes on 17q from 35 Mb to 40 Mb (colored as per panel (B)). (E) Fisher’s exact FDR *p* values for co-amplification of genes with HER2 in 1971 tumors from TCGA and Metabric cohorts. Amplification was defined as 4 or more ploidy-corrected copies. Chromosomes are colored alternatingly in black and gold. (F, G) We detected two regions with germline micro-deletions or micro-gains in normal breast tissue from TCGA: 34.4–34.6 Mb and 44.1–44.8 Mb. These regions are defined as loci with copy number levels either >2.4 or <1.6 in at least 5% of normal breast samples, and were removed from Fig. 2b for visual purposes. Shown are copy number levels in 765 matched normal breast samples, for all genes on chromosome 17, before (F) and after (G) removal of those two regions. (PDF 969 kb)

Additional file 10: HER2 co-amplification events: 14 regions with 931 genes are significantly co-amplified with HER2 in TCGA and Metabric combined cohorts (Fisher’s exact FDR *p* value <0.05). Percentage of amplification in the HER2A vs. non-HER2A tumors, Fisher’s exact FDR *p* value, and gene annotation are shown. Genes in the core or broad HER2 amplicon are listed, and known cancer genes as reported previously [44, 45] are highlighted. (XLSX 104 kb)

Additional file 11: HER2 amplification and mutation in other cancers. (A-C) For each cancer, tumors are grouped by HER2 amplification and mutation status. Tumors without HER2 amplification or mutation are shown in black, HER2A tumors (regardless of HER2 mutation) in red, unamplified tumors with an activating HER2 mutation (HER2MUT act) in green, and unamplified tumors with an untested or functionally inactive HER2 mutation (HER2MUT unk) in gold. (A) Phospho-ERBB3 (Tyr1289) levels are significantly higher in HER2A breast (*p* = 1e-7), endometrium (*p* = 0.003), and lung squamous cell carcinoma (*p* = 0.009) compared to unaltered tumors. (B) Phospho-AKT (pan-AKT Ser473) levels do not differ by HER2 status (*p* = 0.41, 0.13, and 0.45 for HER2A tumors, tumors with an activating HER2 mutation, or a non-functional HER2 mutation, respectively, in comparison to unaltered tumors). (C) Phospho-p44/42 MAPK (ERK1/2) (Thr202/Tyr204) levels do not differ by HER2 status (*p* = 0.26, 0.99, and 0.30, respectively, as shown in (B)). (D) Prevalence of HER2 mutations varies from 0.4 to 8.6%. (PDF 214 kb)

Additional file 12: HER2A tumors in other cancers share certain similarities with breast cancer. (A) Copy number of genes on chromosome 17 are shown in 98 HER2A non-breast tumors. Three distinct groups of HER2A tumors are labeled on the right: tumors with 17q arm-level amplification, defined as 5 or more copies for at least 80% of genes (cyan); tumors with 17q gain (copy number between 2.5 and 5 for 80% or more genes) (magenta); and tumors that are mainly 17q diploid with copy number <2.5 for the majority of 17q genes (green)", with "as per Fig. 2b". Chromosome 17 annotation is indicated on top. Regions 34.4–34.6 Mb and 44.1–44.8 Mb with germline micro-deletions or micro-gains were removed for visual purposes (see Additional file 9F-G). (B) HER2 amplification is a discrete event across multiple molecular subtypes, for cancers with well-established subtypes. HER2-amplified gastric tumors are either chromosomal instable or EBV-positive. (C) Shown on the left axis are the percentage of 98 HER2A non-breast tumors with gene amplification (indicated in solid red) and the percentage of 106 HER2A breast tumors with gene amplification (dashed red), for genes on chromosome 17 from 35 Mb to 40 Mb (ordered by genomic location). Shown on the right axis are the average copy number level in HER2A non-breast tumors (indicated in solid blue) and in HER2A breast tumors (dashed blue) with gene amplification. Shown at the bottom are core pan-cancer HER2 amplicon (6) and broad amplicon (19) genes. (D) 17q12-21 genes that are significantly associated with HER2A in breast cancer (Additional file 4D) show consistent increased expression in HER2A tumors, regardless of cancer (*p* = 1e-235). (E) The three SCGB genes on 11q13 trend towards increased expression in cervix and ovarian cancer, but lack consistent pan-cancer association with HER2A (*p* = 0.72). (D, E) Fold change values were calculated as the difference between log2 average expression, with significance defined as *t* test *p* < 0.05. *P* values were obtained from multivariate linear models, predicting gene set expression by HER2A and cancer in 2838 non-breast tumors. (PDF 271 kb)

Additional file 13: Coordinated expression of HER2-neighboring genes in the absence of amplification. (A-H) Expression of genes in the core HER2 amplicon (*PGAP3*, *ERBB2*, *MIEN1*, *GRB7*), representative genes in the broad HER2 amplicon (*MED1*, *CDK12*, *NR1D1*), and *TOP2A* (more telomeric on 17q), in HER2A tumors (red), non-HER2A tumors without HER2 overexpression (black), and non-HER2A tumors with HER2 overexpression (o/e, log2 nRPKM + 1 ≥ 8.2; green). (A) Seven non-HER2A, o/e breast tumors. (B) Six non-HER2A, o/e gastric tumors. (C) Two non-HER2A, o/e endometrial tumors. (D) Two non-HER2A, o/e cervix tumors. (E) Two non-HER2A, o/e bladder tumors. (F) Two non-HER2A, o/e lung squamous cell carcinoma tumors. (G) One non-HER2A, o/e lung adenocarcinoma tumor. (H) One non-HER2A, o/e ovarian tumor. (I) Relative copy number levels for broad HER2 amplicon genes in 23 non-HER2A o/e tumors. Relative copy number levels exceed 2 (or are borderline at 1.9) in 6 out of 23 tumors. Tumors are colored by cancer, and genes in the broad HER2 amplicon are colored as per Fig. 2a. (J-M) Average log2 ratio of methylated to unmethylated intensity of CpG probes near HER2 and its closest neighbors PGAP3, MIEN1 and GRB7 (in the gene body or maximum 2 kb upstream of the transcription start site) with Kruskal-Wallis test FDR *p* value below the indicated value for a four-group comparison: HER2A, non-o/e; HER2A, o/e; non-HER2A, non-o/e; non-HER2A, o/e. (J) For breast cancer, included are 27 methylation probes with Kruskal-Wallis FDR *p* < 1e-15. (K) For gastric cancer, included are 37 methylation probes with *p* < 1e-5. (L) For cervix cancer, included are 28 methylation probes with *p* < 0.01. (M) For bladder cancer, included are 21 methylation probes with *p* < 0.01. (PDF 571 kb)

## Abbreviations

AKT1: AKT serine/threonine kinase 1
AR: androgen receptor
ASCO: American Society of Clinical Oncology
BRCA1: Breast cancer gene 1 DNA repair associated
CAP: College of American Pathologists
CCNB1IP1: Cyclin B1 interacting protein 1
CIN: Chromosomal instability
CMS: Consensus molecular subtypes
COSMIC: Catalogue of somatic mutations in cancer
CpG: Cytosine base followed immediately by a guanine base
DNA: Deoxyribonucleic acid
EGA: European genome-phenome archive
ER: Estrogen receptor
ERBB2: Erb-b2 receptor tyrosine kinase 2
ERBB3: Erb-b2 receptor tyrosine kinase 3
FDR: False discovery rate
FISH: Fluorescent in-situ hybridization
GATA3: GATA binding protein 3
GATK: Genome analysis toolkit
HER2: Human epidermal growth factor receptor 2 (a synonym for ERBB2)
HER2A: HER2 amplified
HER2E: HER2-enriched
HER2MUT_act: HER2 unamplified tumors with an activating HER2 mutation
HER2MUT_unk: HER2 unamplified tumors with an untested or functionally inactive HER2 mutation
IHC: Immunohistochemistry
IntClust: Integrative cluster
ISH: In situ hybridization
JG: Jiang-Gentleman gene set enrichment score
MAPK: Mitogen-activated protein kinase
nRPKM: Reads per kilobase of exon model per million mapped reads normalized by size factor
NSCLC: Non-small cell lung carcinoma
pAKT: Phosphorylated AKT1/2/3 protein
PAM50: Prediction analysis of microarray 50
pERK: Phosphorylated ERK1/2 protein
pHER2: Phosphorylated HER2 protein
pHER3: Phosphorylated ERBB3 protein
PIK3CA: Phosphatidylinositol-4,5-bisphosphate 3-kinase catalytic subunit alpha
PR: Progesterone receptor
RNA: Ribonucleic acid
RPPA: Reverse phase protein array
SCGB1D2: Lipophilin B
SCGB2A2: Mammoglobin A
SNP: Single nucleotide polymorphism
TCGA: The Cancer Genome Atlas
T-DM1: Trastuzumab emtansine
TN: Triple negative
USO1062: United States oncology trial 1062
