# Supplementary material for: HER2 is not a cancer subtype but rather a pan-cancer event and is highly enriched in AR-driven breast tumors
Source: Breast Cancer Res. 2018 Jan 30;20:8. doi: 10.1186/s13058-018-0933-y (PMC5791377; doi:10.1186/s13058-018-0933-y)
Supplement: Supplementary file 2 — Robust development of the CN/ploidy definition, and comparison with two alternative HER2A measures. (PDF 115 kb) [file 13058_2018_933_MOESM2_ESM.pdf]

Additional File 2: Robust development of the CN/ploidy definition, and comparison with two alternative HER2A measures

We defined an optimal threshold for the ratio of HER2 copy number to tumor ploidy, by assessment of the concordance of CN/ploidy with HER2 over-expression, HER2 IHC/FISH clinical status, HER2 protein abundance, and phosphorylated HER2 protein abundance, in the TCGA and Metabric cohorts. We valued sensitivity (recall) and precision more than specificity, that is, the ability to capture tumors that over-express HER2, that are HER2+ by IHC/FISH, and/or that abundantly express HER2 protein or phosphorylated HER2 levels. We therefore maximized the F1 score, which is defined as 2x precision x recall / (precision + recall), and only depends on positive prediction (true positives, false negatives, false positives). The figure in this document shows ROC curves for the various comparisons with CN/ploidy. Indicated in red is the CN/ploidy threshold at which F1 score reached its maximum. For the TCGA cohort, the optimal threshold equals 2.1 for concordance with clinical HER2 status and HER2 protein abundance, and 2.5 for concordance with mRNA over-expression and pHER2 protein abundance. For the Metabric cohort, the optimal threshold is consistently 2.0.

To guide selection of one of the two thresholds (2 vs. 2.5), the table in this document shows F1 score, accuracy, sensitivity and specificity for both thresholds. Given that 2 was the optimal threshold for 4/6 comparisons, and with only an F1 score difference of 2.1-2.8% for the other two comparisons, we selected 2 as the optimal threshold to distinguish HER2-amplified from non-HER2A tumors based on HER2 CN/ploidy.

We compared the performance of the CN/ploidy definition with two alternative measures of HER2A: 5 or more total copies of HER2, and 4 or more centromere-corrected copies of HER2. For the CN/17centromere definition, we used the average number of copies of all 431 genes on 17p as a surrogate for the centromere's copy number level. This HER2A measure resulted in many false positives (i.e., tumors labeled as HER2A but with low HER2 expression, clinically non-HER2, low HER2 protein, or low phospho-HER2 levels). In TCGA, we observed that the CN/17centromere surrogate was lower than ploidy for 59% of tumors, resulting in a relatively larger fraction of breast tumors considered HER2A. In Metabric, the CN/17centromere surrogate was substantially lower in false positive tumors compared to true HER2A tumors, suggesting that the copy number profile of the 17p arm impacts HER2A classification undesirably.

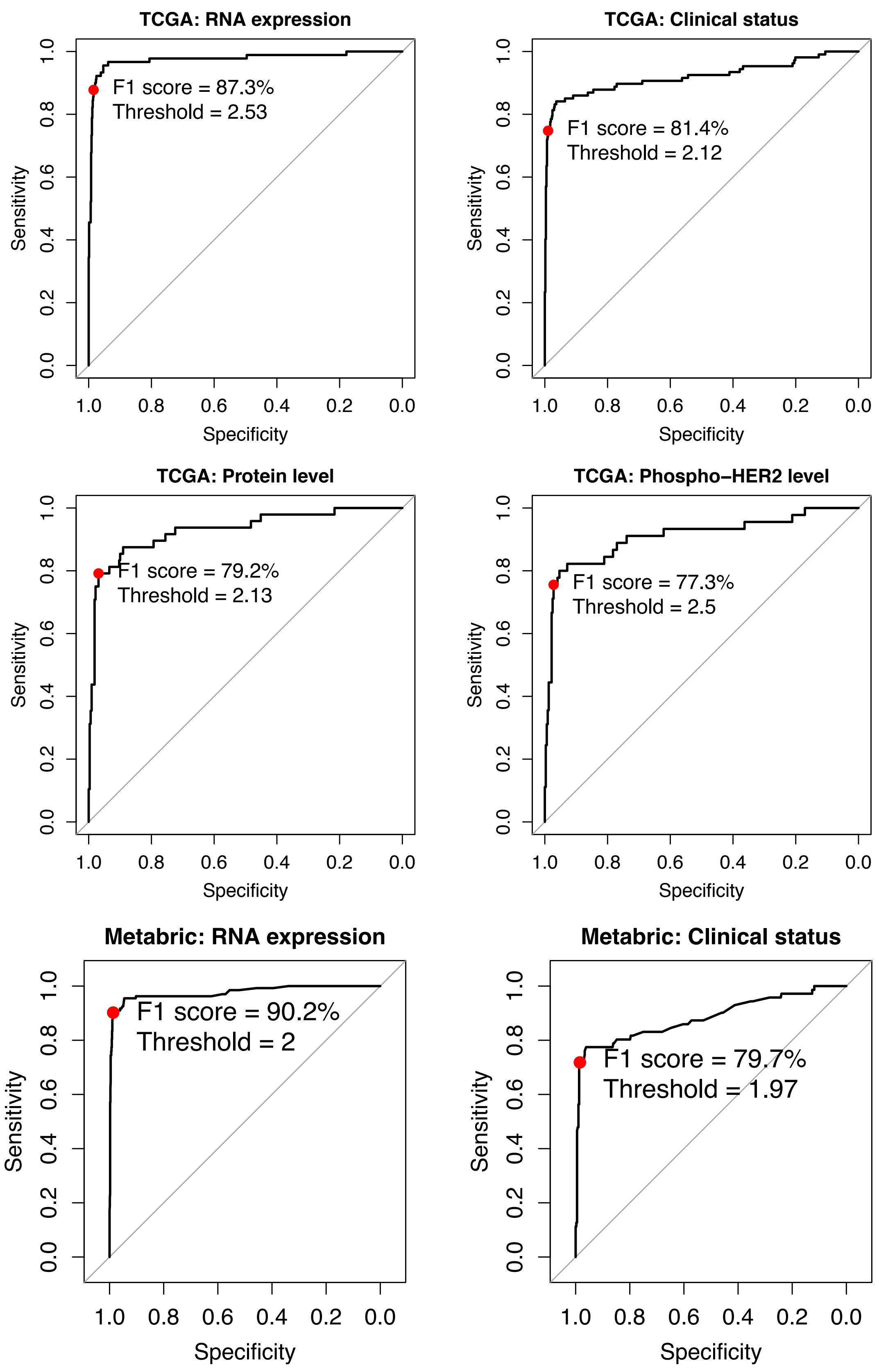
TCGA: RNA expression
F1 score = 87.3%
Threshold = 2.53
TCGA: Clinical status
F1 score = 81.4%
Threshold = 2.12
TCGA: Protein level
F1 score = 79.2%
Threshold = 2.13
TCGA: Phospho−HER2 level
F1 score = 77.3%
Threshold = 2.5
Metabric: RNA expression
F1 score = 90.2%
Threshold = 2
Metabric: Clinical status
F1 score = 79.7%
Threshold = 1.97
Sensitivity
Specificity
0.0
0.2
0.4
0.6
0.8
1.0

Figure: ROC curves for the concordance of HER2 CN/ploidy with HER2 over-expression, HER2 IHC/FISH clinical status, HER2 protein abundance, and phosphorylated HER2 protein abundance, in the TCGA and Metabric cohorts. Labeled is the threshold with maximal F1 score.

Table: F1 score, accuracy, sensitivity and specificity of the prediction of HER2 over-expression, clinical status, protein abundance and/or phosphorylated HER2 levels based on HER2 CN/ploidy in the TCGA and Metabric cohorts, for thresholds 2.0 and 2.5.

**TCGA**	**CN / ploidy threshold**	**F1 score**	**Accuracy**	**Sensitivity**	**Specificity**
**RNA expression**	2.0	84.7	96.5	92.2	97.0
	2.5	86.8	97.2	87.8	98.3
**Clinical status**	2.0	80.1	94.9	74.8	98.6
	2.5	78.3	94.4	68.2	99.3
**Protein level**	2.0	78.4	94.3	79.2	96.6
	2.5	79.1	94.8	75.0	97.8
**Phospho-HER2 level**	2.0	74.5	93.5	77.8	95.7
	2.5	77.3	94.6	75.6	97.2

**Metabric**	**CN / ploidy threshold**	**F1 score**	**Accuracy**	**Sensitivity**	**Specificity**
**RNA expression**	2.0	90.2	97.7	90.2	98.7
	2.5	81.9	96.2	71.4	99.6
**Clinical status**	2.0	78.7	94.1	70.4	98.5
	2.5	69.6	92.4	56.3	99.0
