# Supplementary material for: HER2 is not a cancer subtype but rather a pan-cancer event and is highly enriched in AR-driven breast tumors
Source: Breast Cancer Res. 2018 Jan 30;20:8. doi: 10.1186/s13058-018-0933-y (PMC5791377; doi:10.1186/s13058-018-0933-y)
Supplement: Supplementary file 3 — Definition of HER2-amplified breast cancer. (A) HER2 overexpression in 864 TCGA tumors is defined as log2 (nRPKM + 1) ≥8.2 (normal mixture modeling). (B) Abundant HER2 protein in 367 TCGA tumors is defined as log2 (RPPA) ≥0.92. (C) Abundant phosphorylated HER2 (Tyr1248) is defined as log2 (RPPA) ≥0.605. (D) Concordance of three HER2A classification schemes with HER2 expression, HER2 protein, phosphorylated HER2, clinical HER2 status and PAM50 subtype in TCGA (Additional file 2). In each case, concordance to HER2 gene expression drops with the alternative measures, from 96.5% to 94.7% for total copies (McNemar test p = 0.002), and to 86.1% for centromere-corrected copies (p = 2e-19). Concordance with HER2 protein levels drops from 94.1% to 92.8% (p = 0.18) and 86.6% (p = 7e-6), respectively. Concordance with pHER2 protein levels drops from 93.6% to 91.7% (p = 0.05) and 85% (p = 2e-7). Concordance with clinical HER2 status drops from 94.9% to 94.0% (p = 0.11) and 86.7% (p = 2e-10). Ploidy-corrected HER2A captures a larger fraction of the PAM50 HER2E subtype (90.4% concordance for HER2E vs. other subtypes) than either total (89.0%, p = 0.025) or centromere-corrected HER2A status (80.0%, p = 2e-19). (E) HER2 overexpression in 1107 Metabric tumors is defined as log2 expression ratio ≥12.5. (F) Concordance of three HER2A classification schemes with HER2 expression, clinical HER2 status and PAM50 subtype in Metabric. The concordance to HER2 gene expression drops from 97.7% to 95.1% for total copies (McNemar test p = 1e-5), and to 90.9% for centromere-corrected copies (p = 5e-17). Concordance with clinical HER2 status drops from 94.1% to 91.5% (p = 3e-3) and 90.9% (p = 2e-6), respectively. Overlap with PAM50 HER2E drops from 88.6% to 86.8% (p = 2e-3) and 82.3% (p = 1e-14). (G) HER2 overexpression in 987 USO1062 tumors is defined as normalized log10 counts ≥1.02. (H) Concordance of HER2A status with HER2 overexpression, clinical HER2 status and PAM50 subtype in the USO1062 c [file 13058_2018_933_MOESM3_ESM.pdf]

A

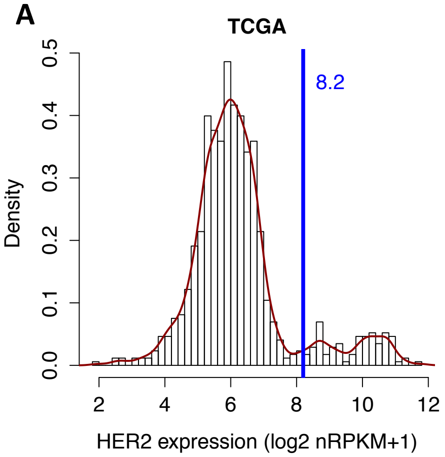

B

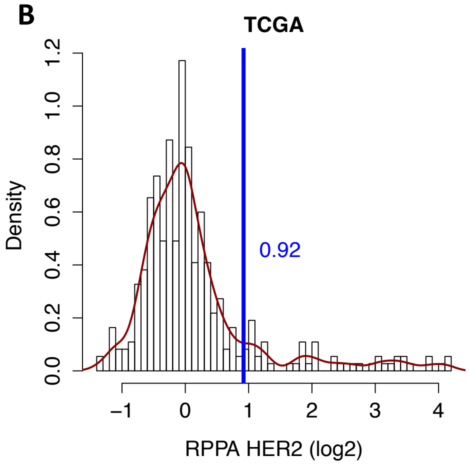

C

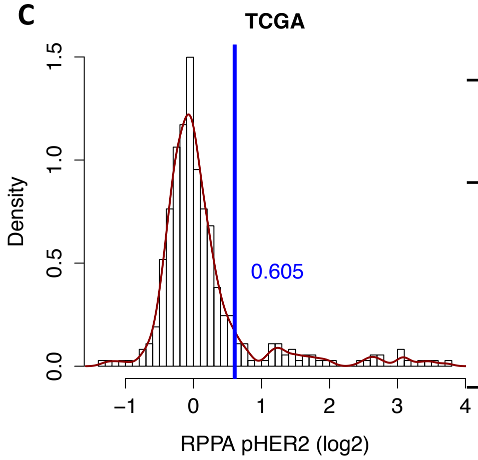

D

TCGA		CN / ploidy HER2A (n=106) n (%)	CN / ploidy Non-HER2A n (%)	CN HER2A (n=118) n (%)	CN Non-HER2A n (%)	CN / 17p HER2A (n=200) n (%)	CN / 17p Non-HER2A n (%)
RNA expression	o/e	83 (78)	7 (0.9)	81 (69)	9 (1.2)	85 (42)	5 (0.8)
	Non-o/e	23 (22)	751 (99)	37 (31)	737 (99)	115 (58)	659 (99)
Protein level	High	38 (78)	10 (3.1)	38 (70)	10 (3.2)	41 (49)	7 (2.5)
	Low	11 (22)	308 (97)	16 (30)	303 (97)	42 (51)	277 (98)
Phospho-HER2 level	High	35 (71)	10 (3.1)	34 (63)	11 (3.5)	36 (43)	9 (3.2)
	Low	14 (29)	308 (97)	20 (37)	302 (96)	47 (57)	275 (97)
Clinical status	Positive	80 (91)	27 (4.5)	80 (85)	27 (4.6)	88 (55)	19 (3.6)
	Negative	8 (9)	568 (95)	14 (15)	562 (95)	72 (45)	504 (96)
PAM50 subtype	HER2E	48 (45)	25 (3.3)	48 (41)	25 (3.3)	50 (25)	23 (3.5)
	Lum A	19 (18)	404 (53)	23 (19)	400 (54)	71 (35)	352 (53)
	Lum B	32 (30)	182 (24)	39 (33)	175 (23)	66 (33)	148 (22)
	Basal	7 (7)	147 (19)	8 (6.8)	146 (20)	13 (6.5)	141 (21)

F

Metabric		CN / ploidy HER2A (n=133) n (%)	CN / ploidy Non-HER2A n (%)	CN HER2A (n=131) n (%)	CN Non-HER2A n (%)	CN / 17p HER2A (n=203) n (%)	CN / 17p Non-HER2A n (%)
RNA expression	o/e	120 (90)	13 (1.3)	105 (80)	28 (2.9)	117 (58)	16 (1.8)
	Non-o/e	13 (10)	961 (99)	26 (20)	948 (97)	86 (42)	888 (98)
Clinical status	Positive	50 (89)	21 (5.2)	42 (81)	29 (7.1)	51 (61)	20 (5.3)
	Negative	6 (11)	382 (95)	10 (19)	378 (93)	33 (39)	355 (95)
PAM50 subtype	HER2E	71 (53)	64 (6.6)	60 (46)	75 (7.7)	71 (35)	64 (7.1)
	Lum A	14 (11)	504 (52)	21 (16)	497 (51)	47 (23)	471 (52)
	Lum B	27 (20)	260 (27)	31 (24)	256 (26)	61 (30)	226 (25)
	Basal	21 (16)	146 (15)	19 (14)	148 (15)	24 (12)	143 (16)

E

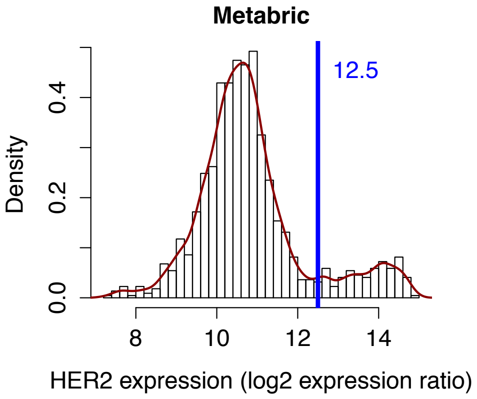

G

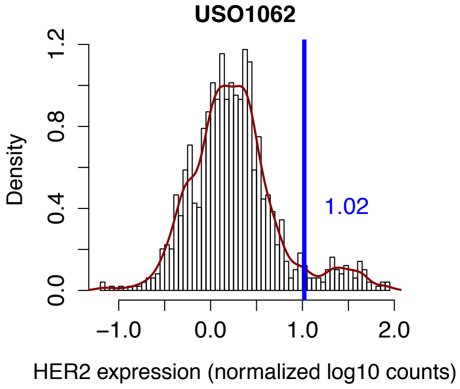

H

USO1062		CN HER2A (n=79) n (%)	CN Non-HER2A n (%)
RNA expression	o/e	58 (73)	11 (1.2)
	Non-o/e	21 (27)	897 (99)
Clinical status	Positive	67 (87)	56 (6.2)
	Negative	10 (13)	849 (94)
PAM50 subtype	HER2E	30 (38)	40 (4.4)
	Lum A	26 (33)	464 (51)
	Lum B	17 (21)	109 (12)
	Basal	6 (8)	295 (32)
