# Supplementary figures and images for: HER2 is not a cancer subtype but rather a pan-cancer event and is highly enriched in AR-driven breast tumors
Source: Breast Cancer Res. 2018 Jan 30;20:8. doi: 10.1186/s13058-018-0933-y (PMC5791377; doi:10.1186/s13058-018-0933-y)

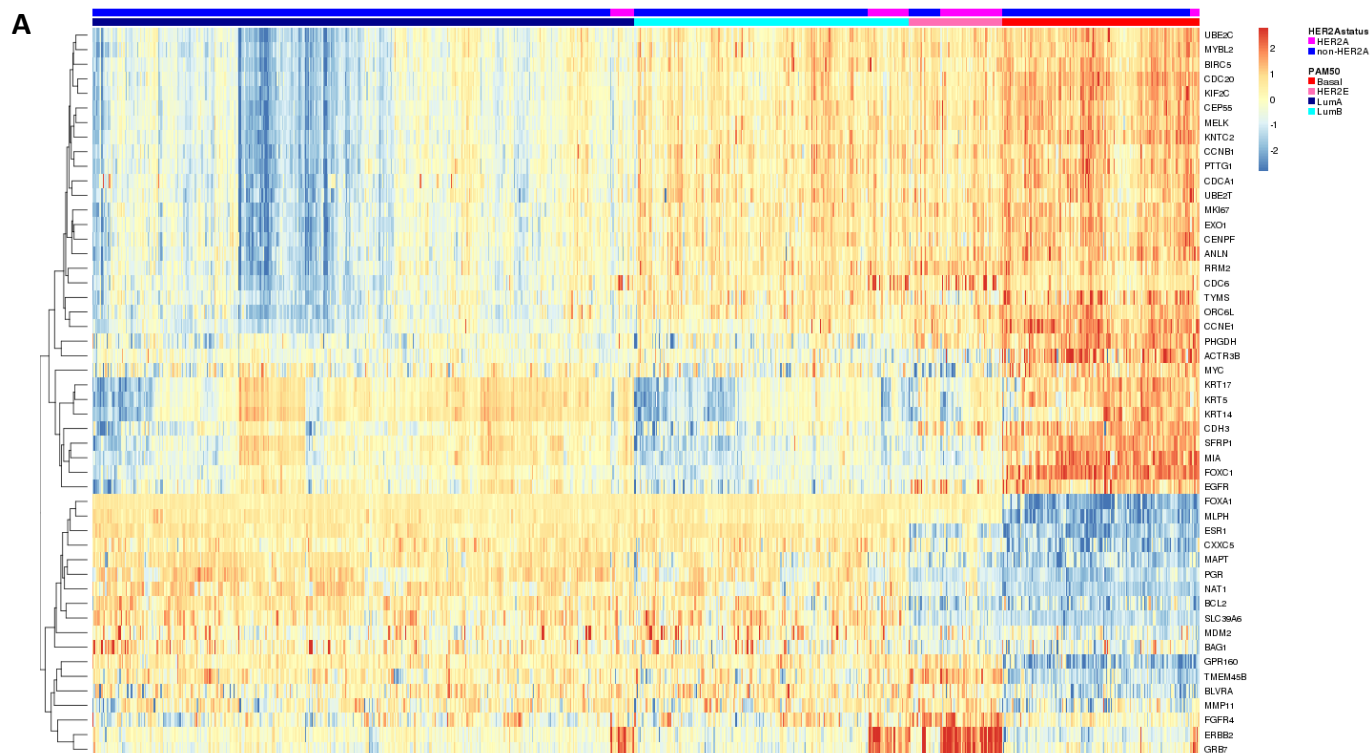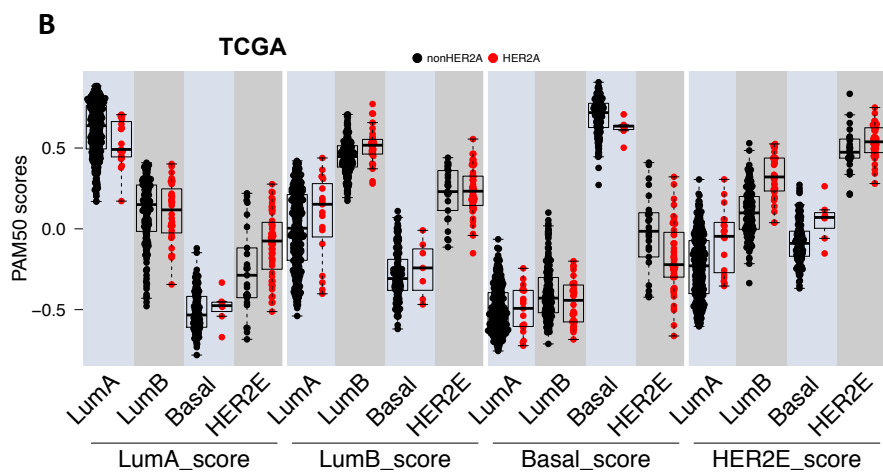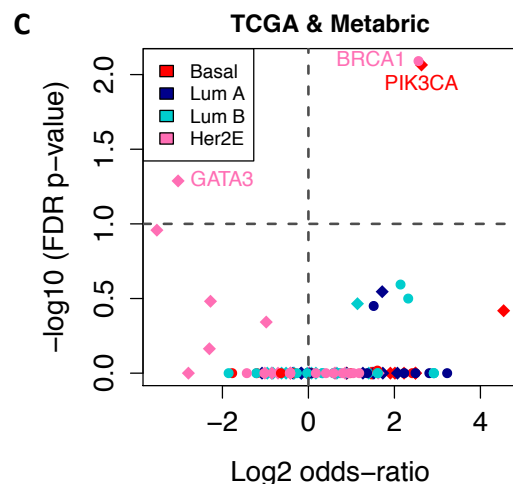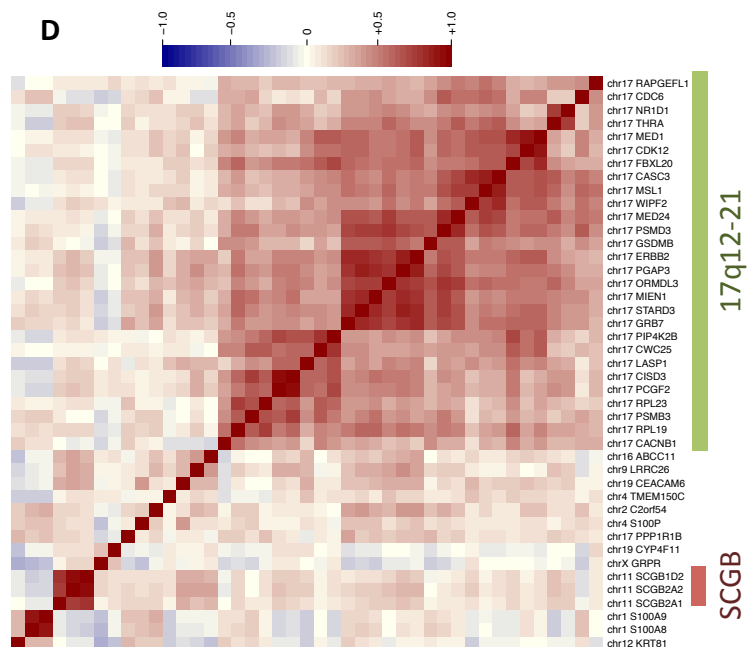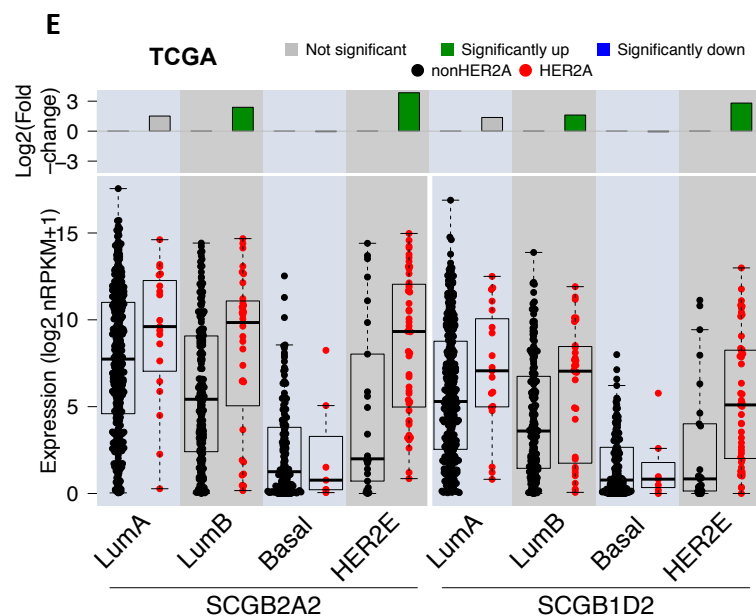

Supplement: Supplementary file 4 — HER2 amplification is a discrete event on top of a luminal or basal state, with minor consistent correlation with gene expression. (A) Row-scaled expression of 50 PAM50 genes in 864 TCGA breast tumors, labeled on top by HER2A status and PAM50 subtype. (B) PAM50 scores for TCGA breast tumors categorized by PAM50 subtype and HER2A status. HER2A tumors, in red, are confidently classified as luminal A, luminal B, basal-like, or HER2E, with PAM50 scores within 3.3–4.3% of the PAM50 scores of non-HER2A tumors of the same subtype. (C) Shown are the odds that a genomic alteration in gene A will occur in an HER2A tumor of subtype X compared to the odds of gene A being altered in a non-HER2A tumor of subtype X. Each dot represents the enrichment of alterations in a gene in HER2A compared to non-HER2A tumors of a particular subtype, colored by PAM50, with mutations shown as diamond and copy number alterations as circle. Fisher’s exact p values were corrected for multiple testing per PAM50 subtype, and separately for the set of 21 genes for which we assessed mutation status (Additional file 5A-B) and 28 genes for which we assessed copy number alterations (Additional file 5C-D). Significance is defined as FDR p value <0.1. Enrichments are based on the combined TCGA and Metabric cohorts. (D) Gene-gene expression correlation in TCGA breast tumors for 43 genes differentially expressed between HER2A and non-HER2A tumors when accounting for PAM50 subtype and chromosomal instability. Two sets of 3 or more highly correlated genes (gene-gene correlation >0.6) are highlighted on the right: 28 genes on 17q12-21 near HER2, and 3 SCGB genes at 11q13 (Additional file 6A). (E) Two genes outside of 17q12-21 are more highly expressed in HER2A than non-HER2A TCGA tumors with FC >4: SCGB2A2 (FC 7.7) and SCGB1D2 (FC 4.4). (PDF 390 kb) [file 13058_2018_933_MOESM4_ESM.pdf]

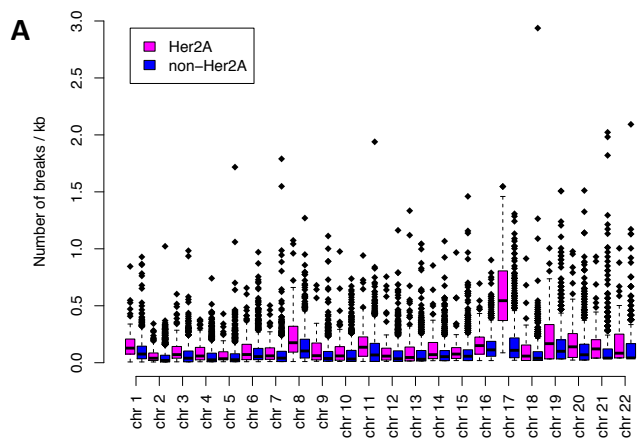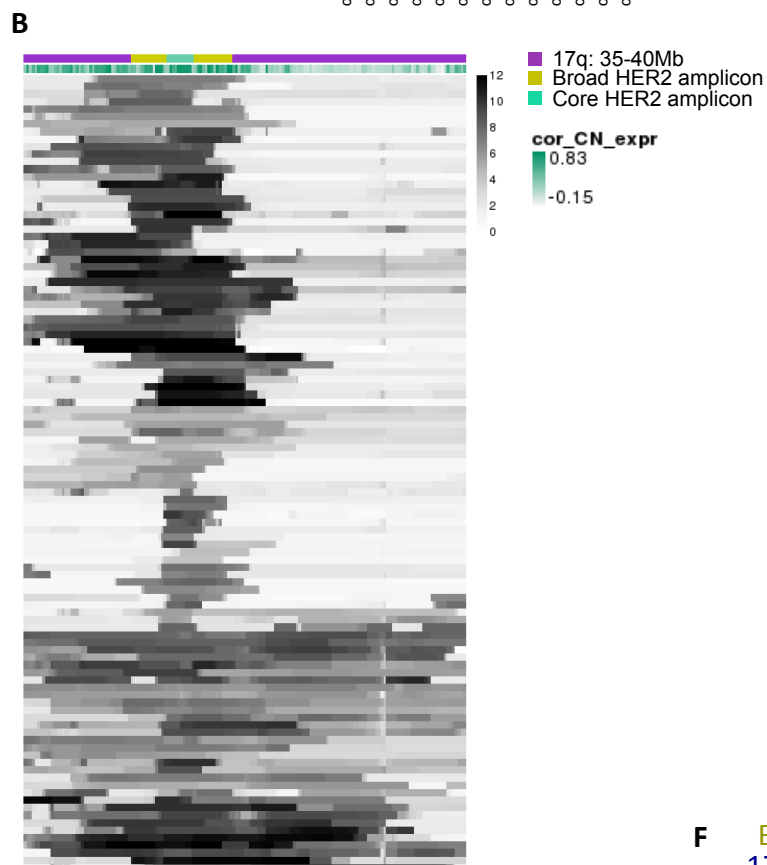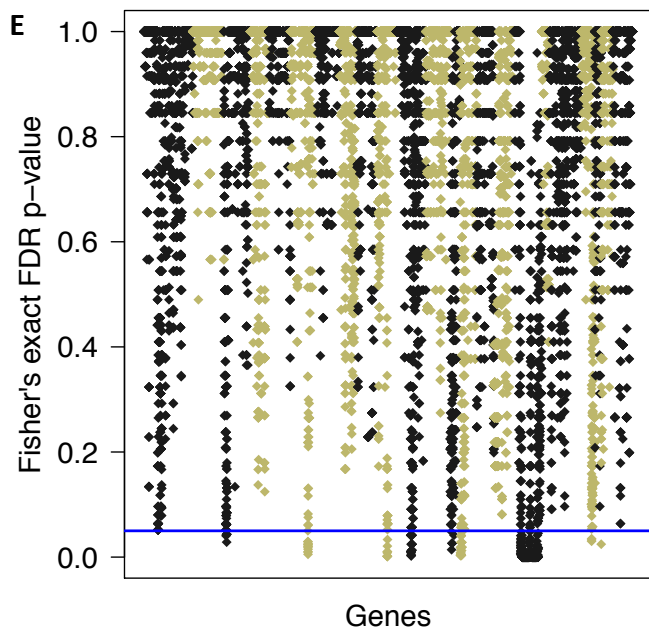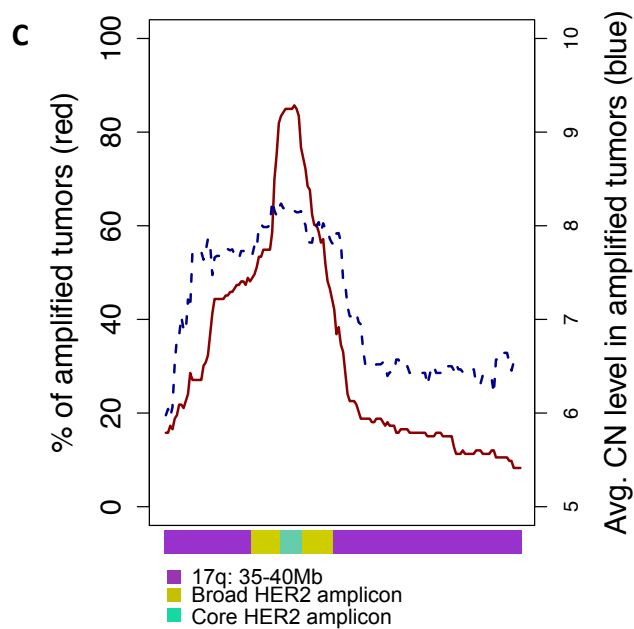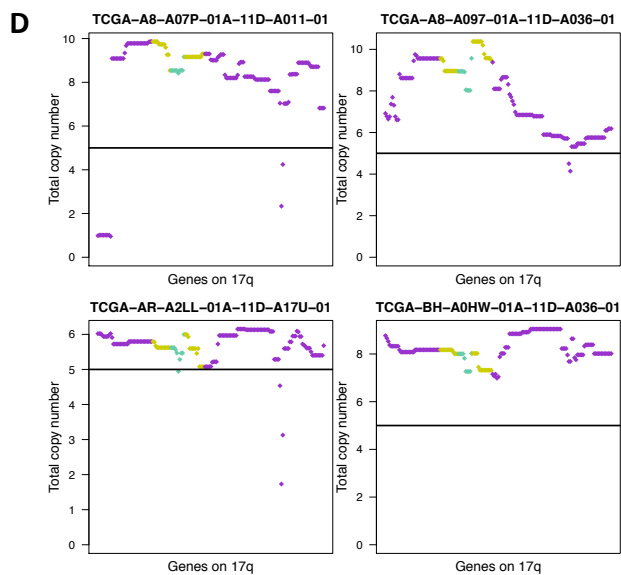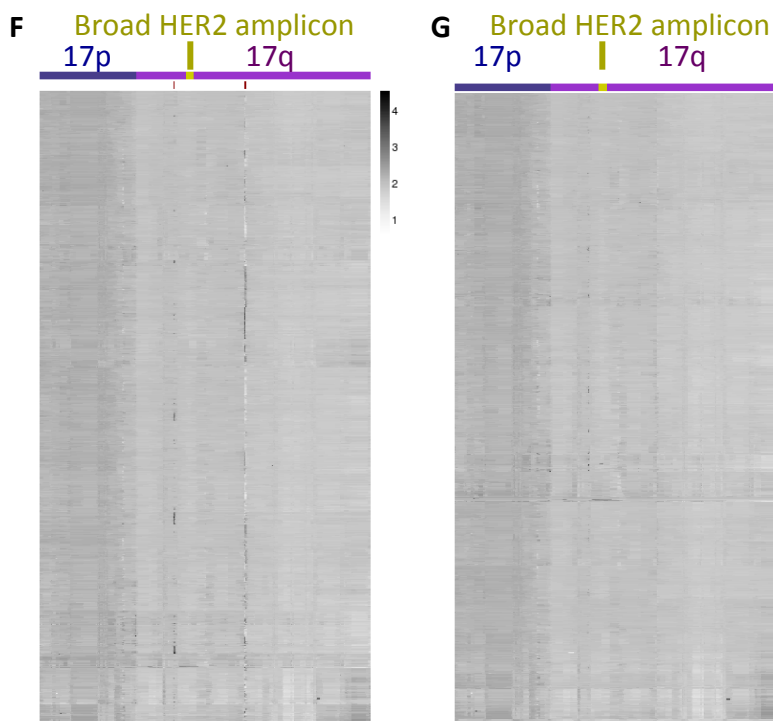

Supplement: Supplementary file 9 — Characterization of the HER2 amplicon and HER2 co-amplification in breast cancer. (A) Chromosomal instability, shown as the number of breaks per kb per autosome, is higher in HER2A than non-HER2A TCGA tumors, with one-sided t test p values per chromosome ranging from 0.37 (chr 5) to 5e-26 (chr 17), and <0.05 for 17/22 autosomes. (B) Copy number levels in 106 HER2A TCGA breast tumors, for genes on chromosome 17 from 35 Mb to 40 Mb (ordered by genomic location). The core HER2 amplicon on top is shown in green, the broad HER2 amplicon in yellow, and genes outside of the broad HER2 amplicon on 17q in magenta. Shown in green on top is correlation between copy number and expression (log2 nRPKM + 1) for each gene across the 864 tumors. (C) HER2 amplicon profile in 133 HER2A Metabric tumors. See Fig. 2a legend for details. (D) Four HER2A TCGA breast tumors show 17q arm-level amplification without additional HER2 focality. Shown are total copy number levels for genes on 17q from 35 Mb to 40 Mb (colored as per panel (B)). (E) Fisher’s exact FDR p values for co-amplification of genes with HER2 in 1971 tumors from TCGA and Metabric cohorts. Amplification was defined as 4 or more ploidy-corrected copies. Chromosomes are colored alternatingly in black and gold. (F, G) We detected two regions with germline micro-deletions or micro-gains in normal breast tissue from TCGA: 34.4–34.6 Mb and 44.1–44.8 Mb. These regions are defined as loci with copy number levels either >2.4 or <1.6 in at least 5% of normal breast samples, and were removed from Fig. 2b for visual purposes. Shown are copy number levels in 765 matched normal breast samples, for all genes on chromosome 17, before (F) and after (G) removal of those two regions. (PDF 969 kb) [file 13058_2018_933_MOESM9_ESM.pdf]

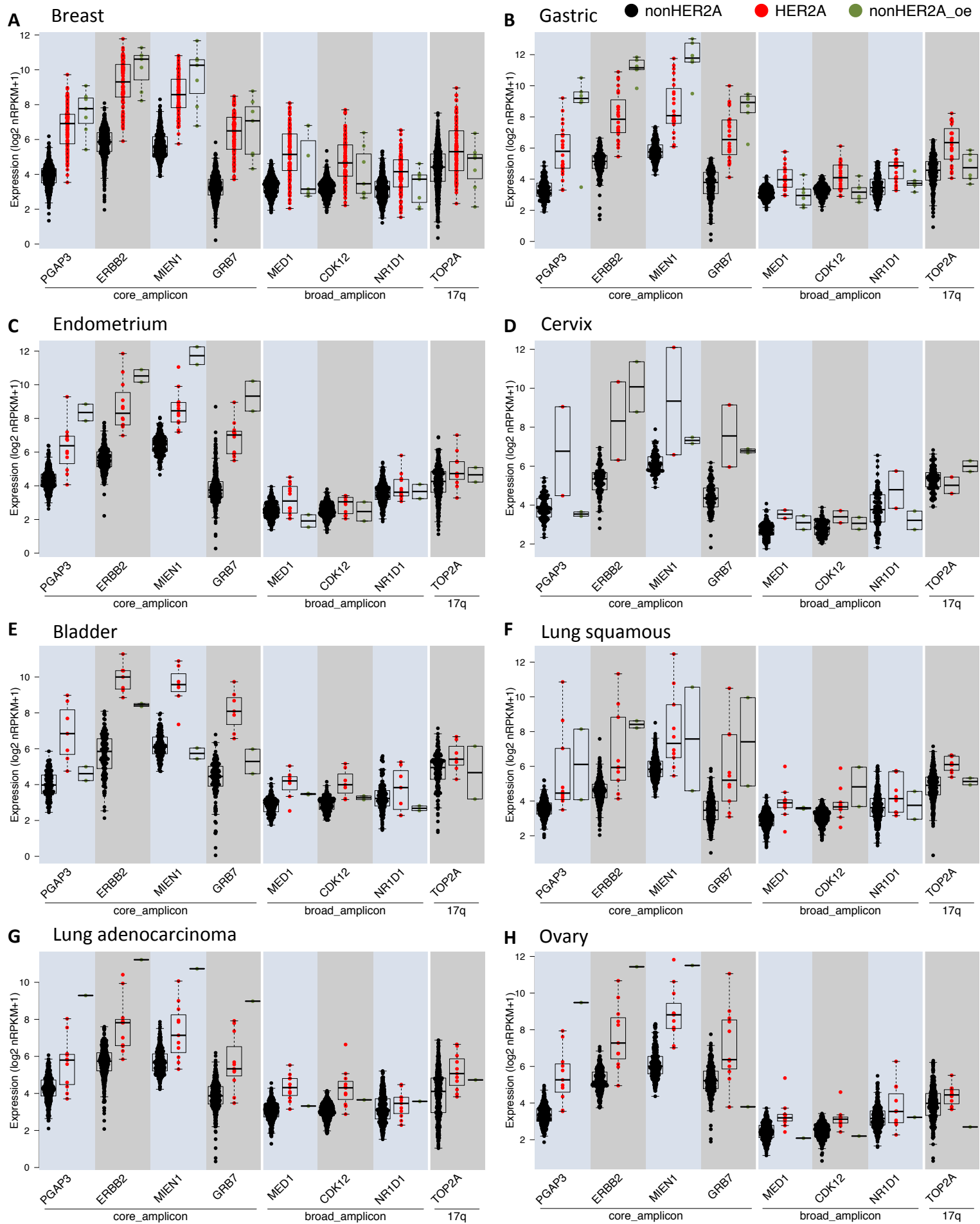

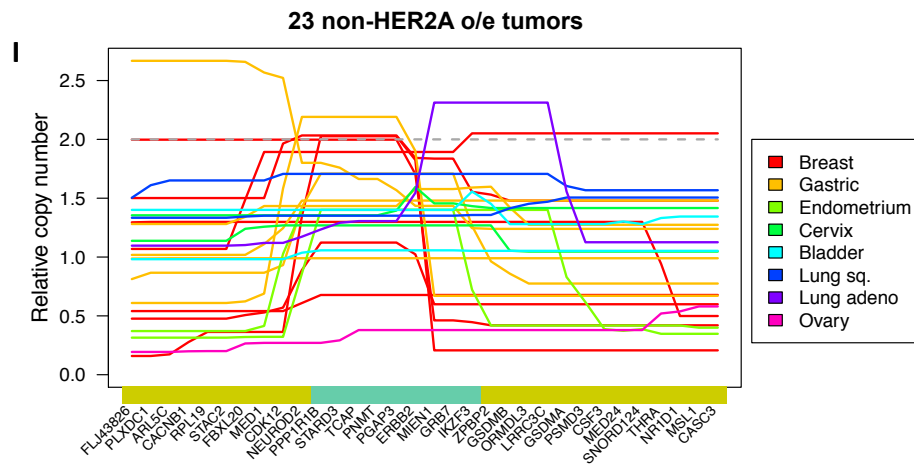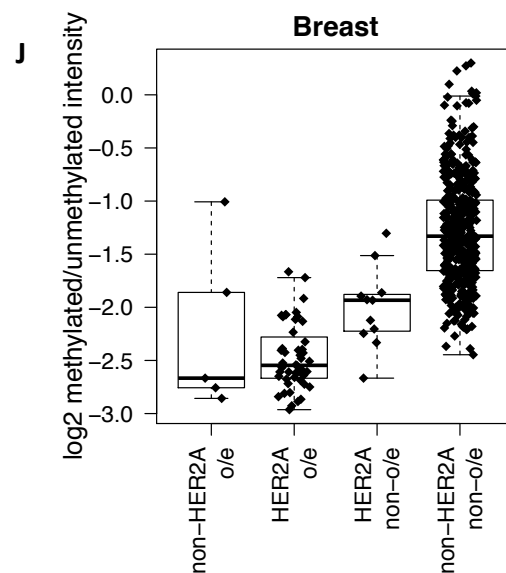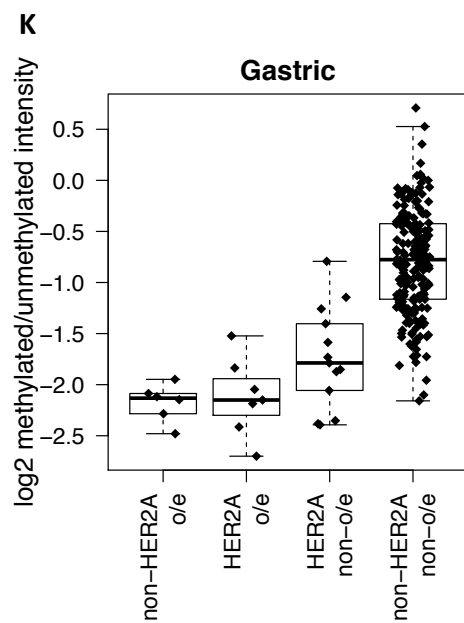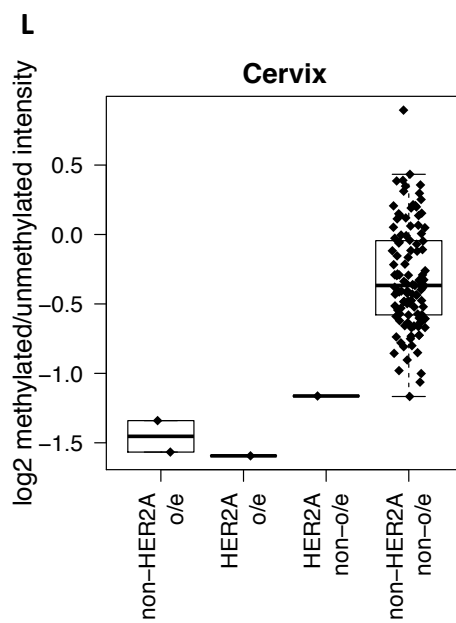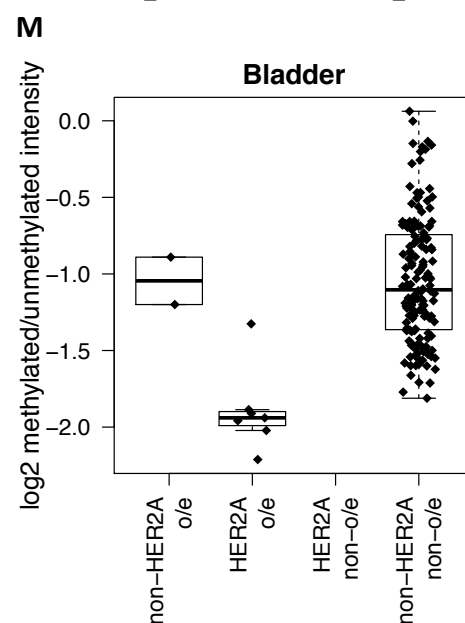

Supplement: Supplementary file 13 — Coordinated expression of HER2-neighboring genes in the absence of amplification. (A-H) Expression of genes in the core HER2 amplicon (PGAP3, ERBB2, MIEN1, GRB7), representative genes in the broad HER2 amplicon (MED1, CDK12, NR1D1), and TOP2A (more telomeric on 17q), in HER2A tumors (red), non-HER2A tumors without HER2 overexpression (black), and non-HER2A tumors with HER2 overexpression (o/e, log2 nRPKM + 1 ≥ 8.2; green). (A) Seven non-HER2A, o/e breast tumors. (B) Six non-HER2A, o/e gastric tumors. (C) Two non-HER2A, o/e endometrial tumors. (D) Two non-HER2A, o/e cervix tumors. (E) Two non-HER2A, o/e bladder tumors. (F) Two non-HER2A, o/e lung squamous cell carcinoma tumors. (G) One non-HER2A, o/e lung adenocarcinoma tumor. (H) One non-HER2A, o/e ovarian tumor. (I) Relative copy number levels for broad HER2 amplicon genes in 23 non-HER2A o/e tumors. Relative copy number levels exceed 2 (or are borderline at 1.9) in 6 out of 23 tumors. Tumors are colored by cancer, and genes in the broad HER2 amplicon are colored as per Fig. 2a. (J-M) Average log2 ratio of methylated to unmethylated intensity of CpG probes near HER2 and its closest neighbors PGAP3, MIEN1 and GRB7 (in the gene body or maximum 2 kb upstream of the transcription start site) with Kruskal-Wallis test FDR p value below the indicated value for a four-group comparison: HER2A, non-o/e; HER2A, o/e; non-HER2A, non-o/e; non-HER2A, o/e. (J) For breast cancer, included are 27 methylation probes with Kruskal-Wallis FDR p < 1e-15. (K) For gastric cancer, included are 37 methylation probes with p < 1e-5. (L) For cervix cancer, included are 28 methylation probes with p < 0.01. (M) For bladder cancer, included are 21 methylation probes with p < 0.01. (PDF 571 kb) [file 13058_2018_933_MOESM13_ESM.pdf]
