# Supplementary material for: HER2 is not a cancer subtype but rather a pan-cancer event and is highly enriched in AR-driven breast tumors
Source: Breast Cancer Res. 2018 Jan 30;20:8. doi: 10.1186/s13058-018-0933-y (PMC5791377; doi:10.1186/s13058-018-0933-y)

**A**

| C2 gene set | Annotation | # genes / gene set size | # sign. genes up / # genes up | # sign. genes down / # genes down | Adj. p-value | JG score |
|---|---|---|---|---|---|---|
| Doane breast cancer classes up | Genes up-regulated in ER-/PR- breast tumors with molecular similarity to ER+, relative to the rest of the ER-/PR- samples | 64/72 | 58/63 | 0/1 | 4e-19 | 58.6 |
| Smid breast cancer ERBB2 up | Genes up-regulated in HER2E tumors (positive control) | 112/147 | 88/110 | 0/2 | 2e-12 | 53.8 |
| Doane breast cancer ESR1 dn | Genes down-regulated in ER+ vs. ER- breast tumors | 44/48 | 2/4 | 35/40 | 5e-12 | -42.5 |
| Farmer breast cancer cluster 6 | Selected luminal genes | 16/16 | 13/16 | 0/0 | 4e-11 | 27.6 |
| Smid breast cancer relapse in brain up | Genes up-regulated in brain relapse of breast cancer, relative to other sites | 36/39 | 0/2 | 24/34 | 6e-8 | -29.7 |
| Farmer breast cancer cluster 7 | Selected apocrine/luminal genes | 18/20 | 15/18 | 0/0 | 2e-7 | 22.3 |
| Doane breast cancer classes dn | Genes down-regulated in ER-/PR- breast tumors with molecular similarity to ER+, relative to the rest of the ER-/PR- samples | 31/34 | 0/1 | 20/30 | 4e-7 | -23.5 |
| Landemaine lung metastasis | Genes up-regulated in lung relapse of breast cancer, relative to other sites | 21/21 | 1/2 | 15/19 | 1e-6 | -24.0 |
| Yang breast cancer ESR1 dn | Genes down-regulated in ER+ vs. ER- breast tumors | 25/25 | 1/2 | 15/23 | 2e-6 | -21.2 |
| Lien breast carcinoma metaplastic vs ductal up | Genes up-regulated in metaplastic vs. ductal breast carcinoma | 70/83 | 1/11 | 37/59 | 5e-6 | -30.0 |

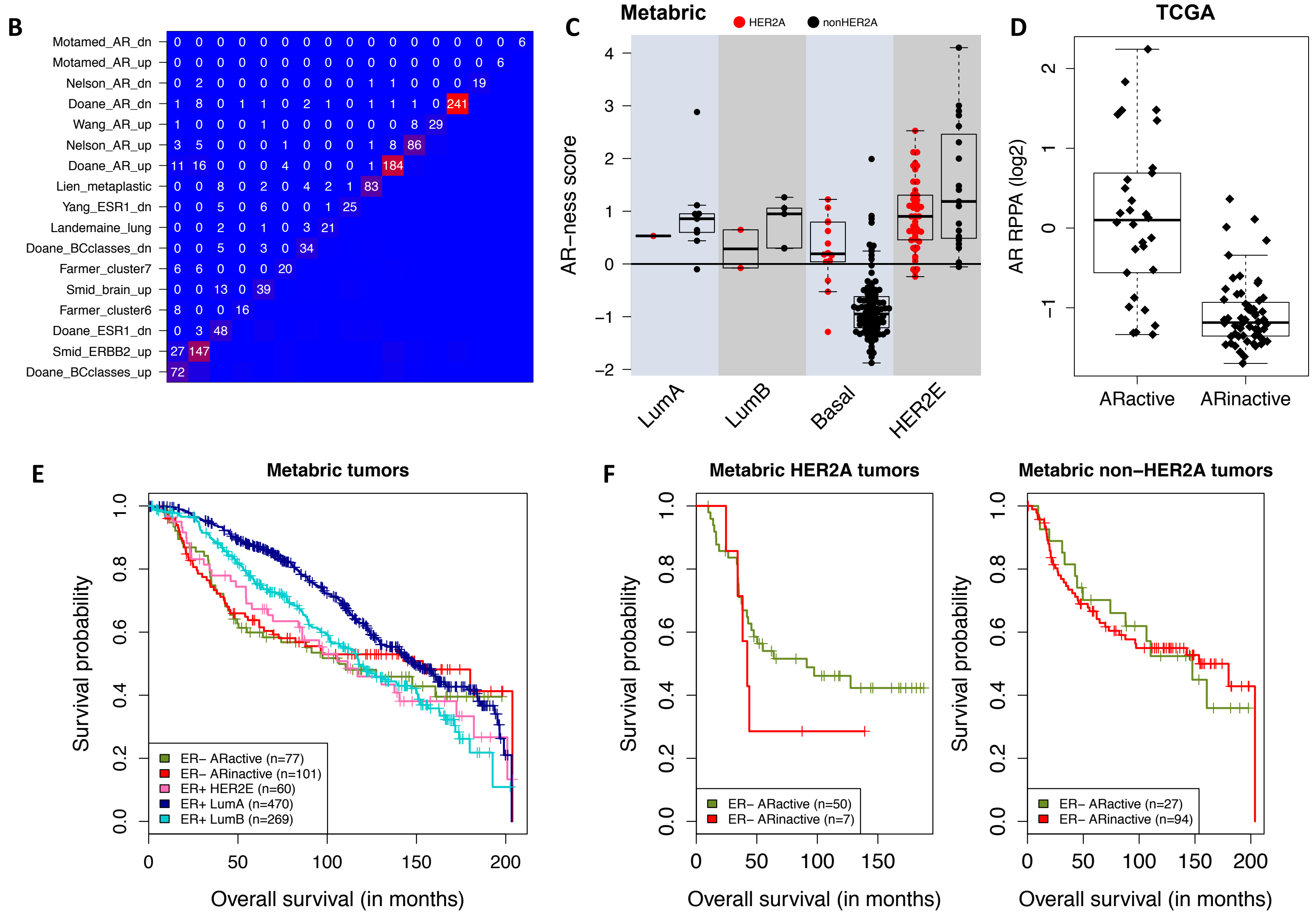

Supplement: Supplementary file 8 — Identification of ER- tumors that are AR-driven. (A) Top 10 C2 gene sets from MSigDB [22] enriched among genes differentially expressed between HER2E and other breast tumors. See Additional file 7B for the full list of C2 gene sets. (B) Overlap in number of genes between the top 10 C2 gene sets and androgen responsive gene sets (from Additional file 7B). (C) AR-ness score across PAM50 subtypes for 205 breast tumors from Metabric that are ER- by IHC. AR-ness score is calculated as the difference in average z-scored expression of 14 positive signature genes and average z-scored expression of 31 negative signature genes. Tumors are colored by HER2A status. (D) AR protein levels in 90 ER- TCGA tumors by AR-ness score. (E) Kaplan-Meier curve of overall survival (OS) in 977 Metabric tumors with a median follow up of 7.3 years, divided into 5 groups based on ER IHC status, PAM50 subtype, and AR activity (positive vs. negative AR-ness score). OS was truncated to 17 years of follow up. (F) Left, Kaplan-Meier curve of OS in 57 ER- HER2A tumors from Metabric, divided by AR-ness score. Right, Kaplan-Meier curve of OS in 121 ER- non-HER2A tumors from Metabric, divided by AR-ness score. OS was truncated to 17 years of follow up. (PDF 167 kb) [file 13058_2018_933_MOESM8_ESM.pdf]
