# Supplementary material for: HER2 is not a cancer subtype but rather a pan-cancer event and is highly enriched in AR-driven breast tumors
Source: Breast Cancer Res. 2018 Jan 30;20:8. doi: 10.1186/s13058-018-0933-y (PMC5791377; doi:10.1186/s13058-018-0933-y)
Supplement: Supplementary file 11 — HER2 amplification and mutation in other cancers. (A-C) For each cancer, tumors are grouped by HER2 amplification and mutation status. Tumors without HER2 amplification or mutation are shown in black, HER2A tumors (regardless of HER2 mutation) in red, unamplified tumors with an activating HER2 mutation (HER2MUT act) in green, and unamplified tumors with an untested or functionally inactive HER2 mutation (HER2MUT unk) in gold. (A) Phospho-ERBB3 (Tyr1289) levels are significantly higher in HER2A breast (p = 1e-7), endometrium (p = 0.003), and lung squamous cell carcinoma (p = 0.009) compared to unaltered tumors. (B) Phospho-AKT (pan-AKT Ser473) levels do not differ by HER2 status (p = 0.41, 0.13, and 0.45 for HER2A tumors, tumors with an activating HER2 mutation, or a non-functional HER2 mutation, respectively, in comparison to unaltered tumors). (C) Phospho-p44/42 MAPK (ERK1/2) (Thr202/Tyr204) levels do not differ by HER2 status (p = 0.26, 0.99, and 0.30, respectively, as shown in (B)). (D) Prevalence of HER2 mutations varies from 0.4 to 8.6%. (PDF 214 kb) [file 13058_2018_933_MOESM11_ESM.pdf]

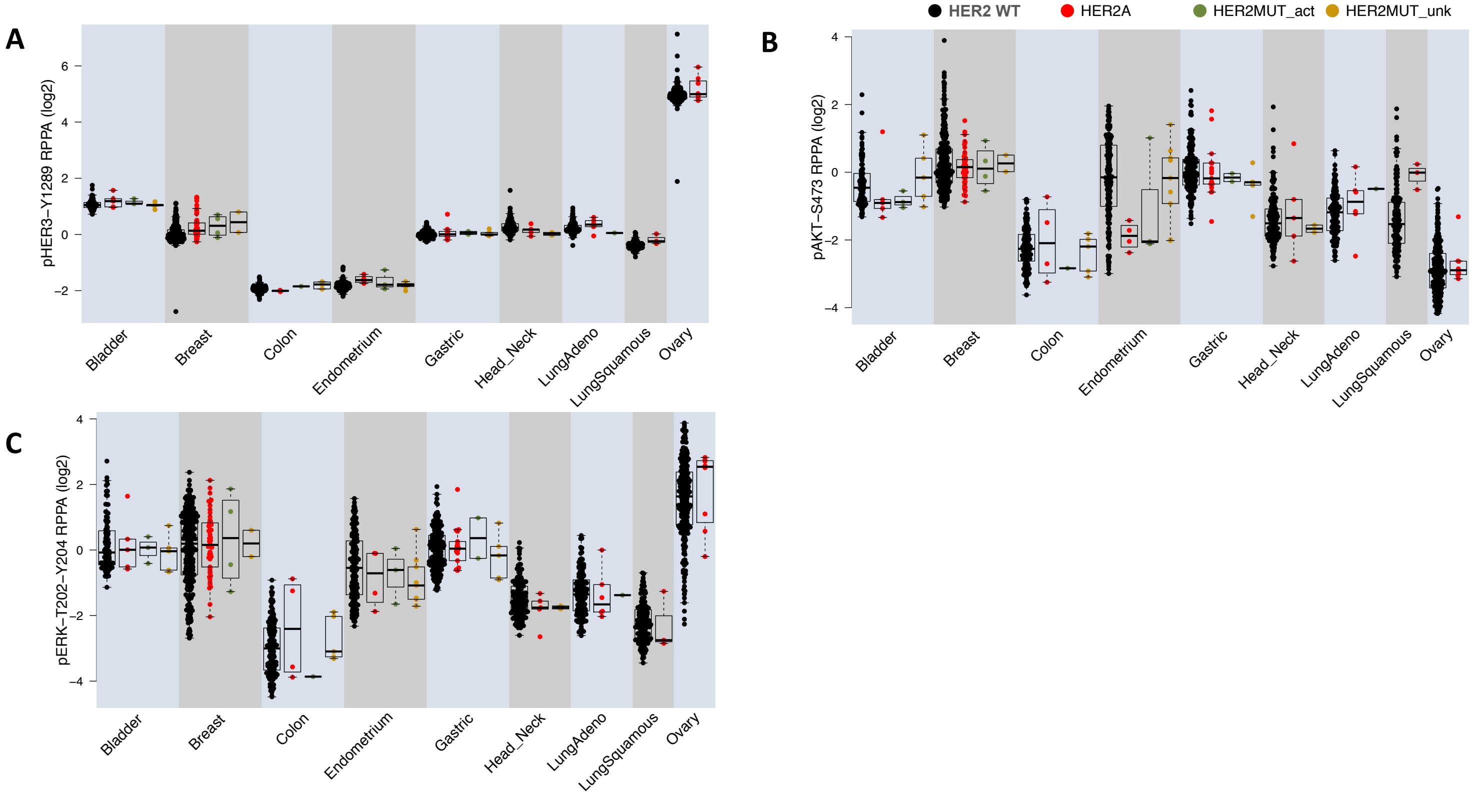

D	Total # patients (%)	# HER2A (%)	# non-HER2A (%)	Fisher's exact test, p-value	Activating HER2 mutations (# tumors)
Breast	21 (3.1)	4 (4.4)	17 (2.9)	0.51	G309A (1), S310F (1), L755S (3), D769H (1), D769Y (2), V777L (1), V842I (1)
Gastric	9 (3.8)	0 (0)	9 (4.1)	1.00	S310F (2), V842I (1)
Bladder	13 (8.6)	2 (28.6)	11 (7.6)	0.11	S310F (4), L755S (1), T862A (1)
Lung Adenocarc.	2 (0.5)	0 (0)	2 (0.5)	1.00	S310F (1)
Lung Sq. cell carc.	4 (1.2)	0 (0)	4 (1.3)	1.00	
Ovary	1 (0.4)	0 (0)	1 (0.4)	1.00	V842I (1)
Colon	7 (4.1)	1 (20)	6 (3.6)	0.19	S310F (1), V777L (1)
Head & Neck	2 (0.6)	0 (0)	2 (0.6)	1.00	
Cervix	3 (3.5)	0 (0)	3 (3.6)	1.00	S310F (2)
Uterine	0 (0)	0 (0)	0 (0)	--	
Endometrium	27 (7.9)	0 (0)	27 (8.2)	1.00	L755S (3), V842I (4)
