# Supplementary material for: HER2 is not a cancer subtype but rather a pan-cancer event and is highly enriched in AR-driven breast tumors
Source: Breast Cancer Res. 2018 Jan 30;20:8. doi: 10.1186/s13058-018-0933-y (PMC5791377; doi:10.1186/s13058-018-0933-y)

A

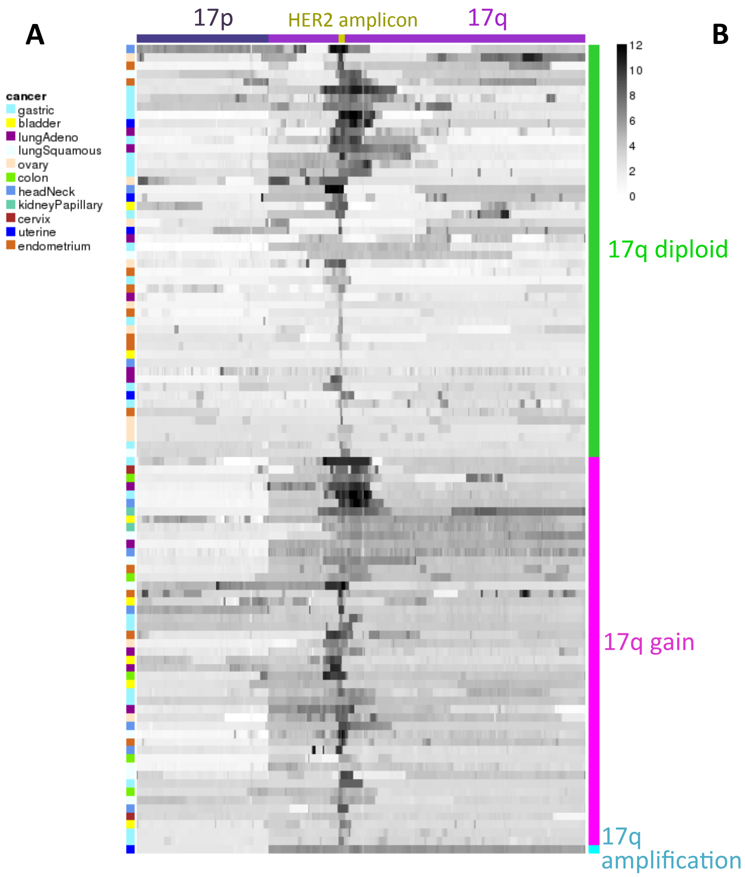

B

		HER2A	Non-HER2A
Gastric	Chromosomal instable	21 (16.4%)	107
	Epstein-Barr virus-positive	3 (12.5%)	21
	Genomically stable	0 (0%)	49
	Microsatellite instable	0 (0%)	58
Bladder	I Papillary-like / luminal	2 (5.1%)	37
	II Luminal	3 (7.9%)	35
	III Basal / squamous-like	2 (7.1%)	26
	IV	0 (0%)	15
Colon	CMS1	0 (0%)	33
	CMS2	3 (4.8%)	60
	CMS3	0 (0%)	32
	CMS4	1 (1.8%)	55
Ovary	Differentiated	4 (3.9%)	99
	Immunoreactive	3 (3.5%)	83
	Mesenchymal	2 (2.2%)	89
	Proliferative	2 (1.8%)	107
Head & Neck	Atypical	2 (3%)	65
	Basal	0 (0%)	82
	Classical	3 (6.1%)	46
	Mesenchymal	1 (1.4%)	70

C

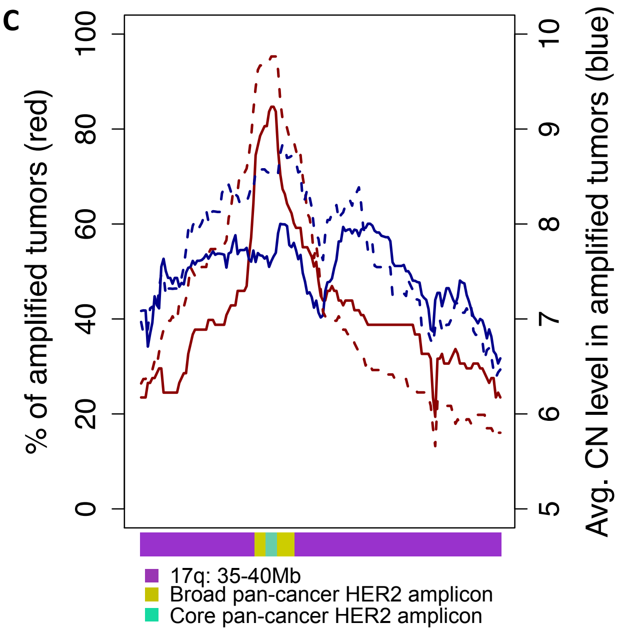

D

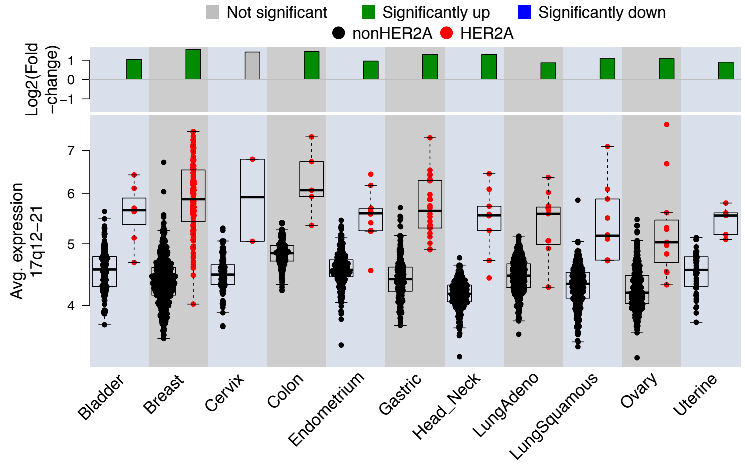

E

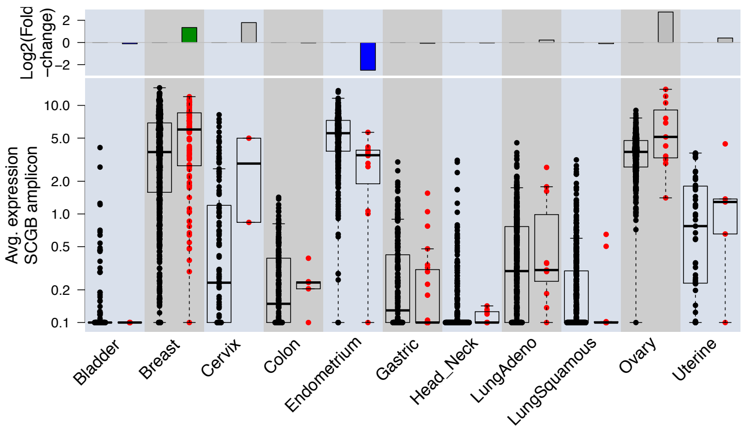

Supplement: Supplementary file 12 — HER2A tumors in other cancers share certain similarities with breast cancer. (A) Copy number of genes on chromosome 17 are shown in 98 HER2A non-breast tumors. Three distinct groups of HER2A tumors are labeled on the right: tumors with 17q arm-level amplification, defined as 5 or more copies for at least 80% of genes (cyan); tumors with 17q gain (copy number between 2.5 and 5 for 80% or more genes) (magenta); and tumors that are mainly 17q diploid with copy number <2.5 for the majority of 17q genes (green)", with "as per Fig. 2b". Chromosome 17 annotation is indicated on top. Regions 34.4–34.6 Mb and 44.1–44.8 Mb with germline micro-deletions or micro-gains were removed for visual purposes (see Additional file 9F-G). (B) HER2 amplification is a discrete event across multiple molecular subtypes, for cancers with well-established subtypes. HER2-amplified gastric tumors are either chromosomal instable or EBV-positive. (C) Shown on the left axis are the percentage of 98 HER2A non-breast tumors with gene amplification (indicated in solid red) and the percentage of 106 HER2A breast tumors with gene amplification (dashed red), for genes on chromosome 17 from 35 Mb to 40 Mb (ordered by genomic location). Shown on the right axis are the average copy number level in HER2A non-breast tumors (indicated in solid blue) and in HER2A breast tumors (dashed blue) with gene amplification. Shown at the bottom are core pan-cancer HER2 amplicon (6) and broad amplicon (19) genes. (D) 17q12-21 genes that are significantly associated with HER2A in breast cancer (Additional file 4D) show consistent increased expression in HER2A tumors, regardless of cancer (p = 1e-235). (E) The three SCGB genes on 11q13 trend towards increased expression in cervix and ovarian cancer, but lack consistent pan-cancer association with HER2A (p = 0.72). (D, E) Fold change values were calculated as the difference between log2 average expression, with significance defined as t test p < 0.05. P values were obtained [file 13058_2018_933_MOESM12_ESM.pdf]
